# Halide Electrolyte Effects in the Electrochemical Hydrogenation of Ketones on Copper

**DOI:** 10.1021/jacsau.6c00452

**Published:** 2026-06-23

**Authors:** Jose Solera-Rojas, Didac A. Fenoll, Elena Segura-Sanchis, M. Consuelo Barrantes, Conor Brennan-Pollak, Francisco Fabregat-Santiago, Carmen Mejuto, Max García-Melchor, Elena Mas-Marzá

**Affiliations:** † Institute of Advanced Materials (INAM), 16748 Universitat Jaume I, 12006 Castelló, Spain; ‡ Center for Cooperative Research on Alternative Energy (CIC energiGUNE), Basque Research and Technology Alliance (BRTA), Vitoria-Gasteiz 01510, Spain; § School of Chemistry, Trinity College Dublin, College Green, Dublin 2, Dublin,Ireland; ∥ IKERBASQUE, Basque Foundation for Science, Plaza de Euskadi 5, 48009 Bilbao, Spain

**Keywords:** electrochemical hydrogenation, copper electrocatalysts, ketone hydrogenation, biomass valorization, halide electrolytes, electrified interfaces, reaction mechanisms, density functional theory

## Abstract

Electrochemical hydrogenation of biomass-derived ketones offers a sustainable route to value-added chemicals but is often limited by competition from the hydrogen evolution reaction (HER). Here, we investigate how electrolyte composition, and specifically halide identity, governs the selective electrochemical hydrogenation of acetophenone to 1-phenylethanol on copper electrodes under near-neutral aqueous conditions. Potassium halide electrolytes introduce specific halide-surface interactions that modulate hydrogen adsorption, delay HER onset, and influence organic reduction behavior. Among the halides studied, chloride- and bromide-containing electrolytes exhibit higher activity and selectivity toward 1-phenylethanol formation than iodide, highlighting a strong dependence of catalytic performance on halide identity. Systematic variation of applied potential, electrolyte identity, halide composition, and acetophenone concentration reveal strong correlations between catalytic performance and electrolyte-dependent interfacial chemistry. Kinetic analysis, hydrogen scavenging experiments, and electrochemical in situ surface-enhanced Raman spectroscopy suggest a surface-mediated hydrogenation pathway involving adsorbed acetophenone and surface-derived hydrogen species, consistent with a Langmuir–Hinshelwood-like mechanism. Density functional theory calculations of hydrogen and halide coadsorption on Cu(111) provide molecular-level insights into these observations, showing that iodide remains more stable on the copper surface at cathodic potentials than chloride or bromide. This persistent iodide coverage limits surface accessibility for acetophenone adsorption, resulting in reduced hydrogenation activity despite delayed HER. Together, these results demonstrate how halide electrolyte identity tunes surface coverage, reaction kinetics, and selectivity during electrochemical hydrogenation, highlighting electrolyte engineering as an effective strategy for enabling selective ketone reduction under mild, near-neutral conditions.

## Graphical abstract

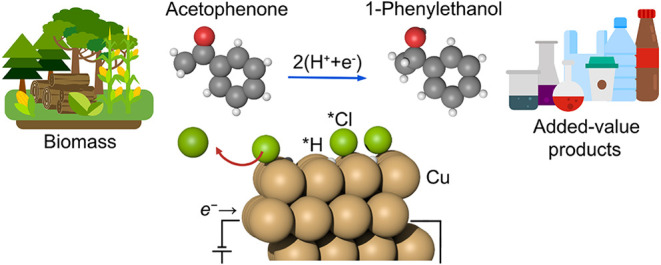

## 1 Introduction

Lignocellulosic biomass, composed primarily of cellulose, hemicellulose, and lignin, represents the most abundant renewable carbon-based feedstock for the sustainable production of chemicals and fuels. Due to the presence of diverse reactive functional groups, including C=C, C=O, and C≡N moieties, biomass-derived intermediates can, in principle, be converted into a wide range of value-added products.^1,2^ Despite significant progress in biomass valorization, efficient and scalable routes for the selective upgrading of these intermediates into platform chemicals remain limited.

Lignin, which constitutes nearly 25% of lignocellulosic biomass, is a highly cross-linked phenolic polymer derived from aromatic building blocks such as benzoic acid, phenol, benzaldehyde, and acetophenone (Figure 1).^3^ These aromatic compounds are versatile molecular precursors that can be transformed into fuels and fine chemicals through hydrogenation, deoxygenation, or decarbonylation pathways.^4^

**Figure 1:**
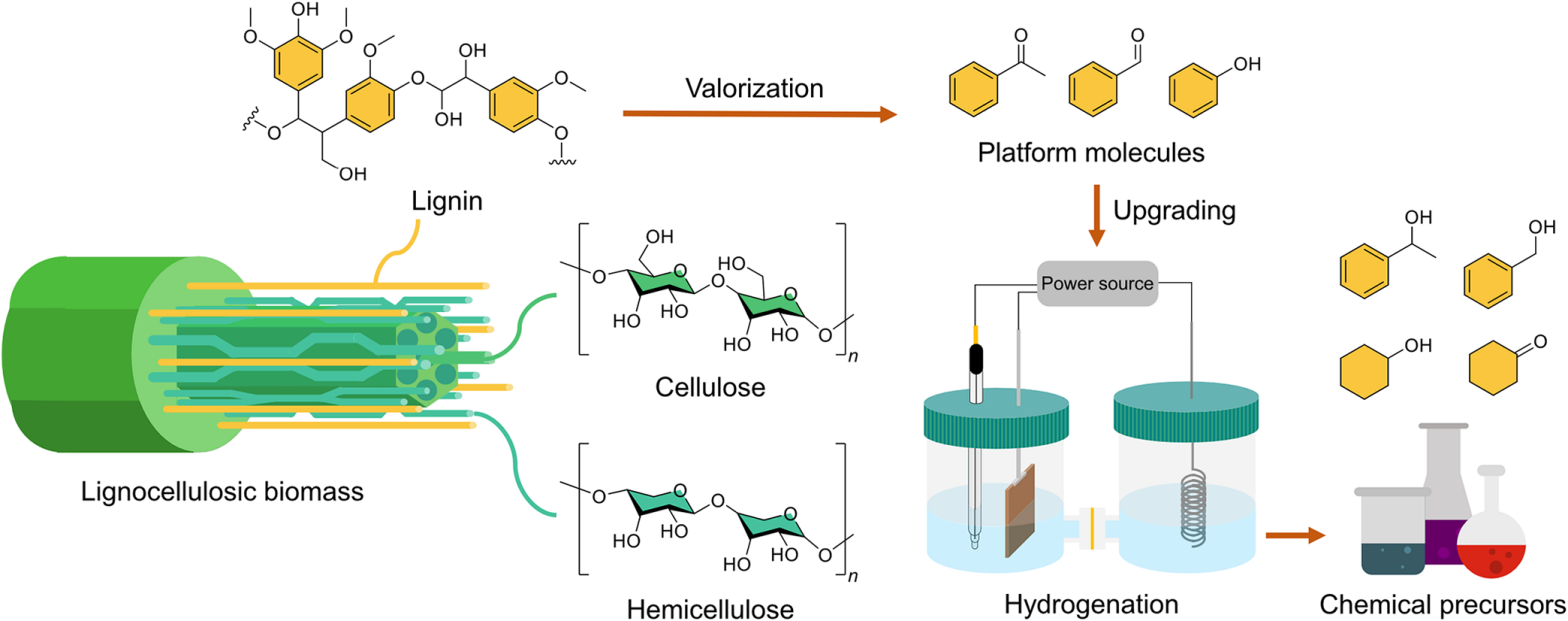
Schematic representation of the valorization and upgrading of lignocellulosic biomass into molecular precursors.

Conventional hydrogenation of lignin-derived aromatics typically relies on thermocatalytic processes that require elevated temperatures and pressures, as well as noble metal catalysts (e.g., Au, Pt, Ru, and Pd).^5,6^ These conditions result in high energy consumption and raise safety and environmental concerns. In contrast, electrochemical hydrogenation (ECH) offers a more sustainable alternative by operating under ambient conditions, using water as the proton source, and enabling direct coupling to renewable electricity.^7–9^ By generating reactive hydrogen species in situ at the electrode–electrolyte interface, ECH eliminates the need for external hydrogen gas or stoichiometric reductants and provides precise control over the applied potential and reaction selectivity.

To date, most ECH studies have focused on aldehydes because of their higher electrophilicity and lower reduction onsets.^10–16^ This focus reflects the intrinsic challenges associated with ketone electrochemical hydrogenation, which remains considerably more demanding. More specifically, steric hindrance and weaker carbonyl activation increase the required overpotential and favor the competing hydrogen evolution reaction (HER), thereby reducing Faradaic efficiency (FE).^17–19^ Controlling the dynamic surface coverage of hydrogen and organic intermediates through careful selection of pH and applied potential has therefore emerged as an effective strategy for improving ECH performance.^20,21^ For example, strongly alkaline conditions reduce surface hydrogen availability, delaying HER and promoting ketone hydrogenation when water-derived hydrogen binds only weakly to the electrode surface.^21,22^ However, highly basic environments can also promote undesired side reactions, including Cannizzaro reactions,^23,24^ aldol condensation,^25^ polymerization,^26^ and C–C coupling,^27^ complicating product control.

Beyond pH effects, recent studies have shown that electrolyte composition can play an equally important role in governing interfacial reactivity during ECH. In particular, halide ions such as chloride have been reported to partially block or restructure metal surfaces, thereby strongly influencing HER kinetics on metal surfaces by altering hydrogen adsorption or surface coverage,^28,29^ and by tuning specific organic reduction pathways.^20,30–33^ Despite these observations, the mechanistic role of halide ions in regulating surface coverage, hydrogen adsorption, and reaction pathways during ketone ECH remains poorly understood.

Copper is an attractive model electrode for addressing these questions because of its intrinsically low HER activity compared to more HER-active transition metals. As previously reported by Ciotti et al.,^21^ the inherently low HER activity of copper favors the selective adsorption and reduction of organic molecules such as acetophenone (ACT) in alkaline media. Beyond its utility as a model ketone, ACT is also an industrially relevant substrate. Its electroreduction yields 1-phenylethanol (PE), a key intermediate for the pharmaceutical, agrochemical, and flavor industries.^34^ Subsequent dehydration of PE produces styrene, an important monomer for polymer production, further underscoring the industrial relevance of this transformation.^35,36^

Herein, we use ACT as a model ketone to investigate electrolyte effects on electrochemical hydrogenation under neutral conditions using copper electrodes. We show that potassium chloride (KCl) and potassium bromide (KBr) electrolytes enable high ACT conversion and PE selectivity, whereas potassium iodide (KI) markedly decreases Faradaic efficiency and PE yield. By combining kinetic analysis, electrochemical in situ surface-enhanced Raman spectroscopy (EC-SERS), scavenger quenching experiments, and density functional theory (DFT) calculations, we elucidate how halide identity governs surface coverage and reaction pathways under neutral conditions. Our results reveal that iodide remains strongly bound to the copper surface under operating conditions, delaying HER but simultaneously hindering ACT adsorption. In contrast, chloride and bromide desorb at less cathodic potentials, allowing earlier ACT adsorption and more efficient hydrogenation. Mechanistic studies were further conducted to evaluate the preference for Langmuir–Hinshelwood (L–H) versus Eley–Rideal (E–R) hydrogenation pathways.^37^ The combined experimental and theoretical results are more consistent with an L–H-like surface-mediated mechanism involving coadsorbed ACT and hydrogen species generated from water dissociation, in agreement with previous theoretical studies.^21^ Together, these findings establish electrolyte engineering, and specifically halide effects, as a powerful strategy for tuning interfacial chemistry and reaction kinetics in electrochemical ketone hydrogenation. These insights highlight the importance of electrolyte–catalyst codesign as a strategy for the rational development of highly selective electrochemical hydrogenation catalysts.

## 2 Results and Discussion

### 2.1 Preparation and Characterization of Copper Electrodes

Copper-based electrodes (hereafter denoted as CuE) were prepared using the dynamic hydrogen bubble template (DHBT) method. In this approach, a porous copper layer is electrodeposited onto a copper foil support, as schematically illustrated in Figure 2a. As reported previously, the concurrent generation of hydrogen bubbles during electrodeposition directs metal growth, promoting strong adhesion to the substrate and the formation of a highly porous morphology.^21,38,39^ Detailed experimental procedures are provided in Section S1.2 of the Supporting Information.

**Figure 2:**
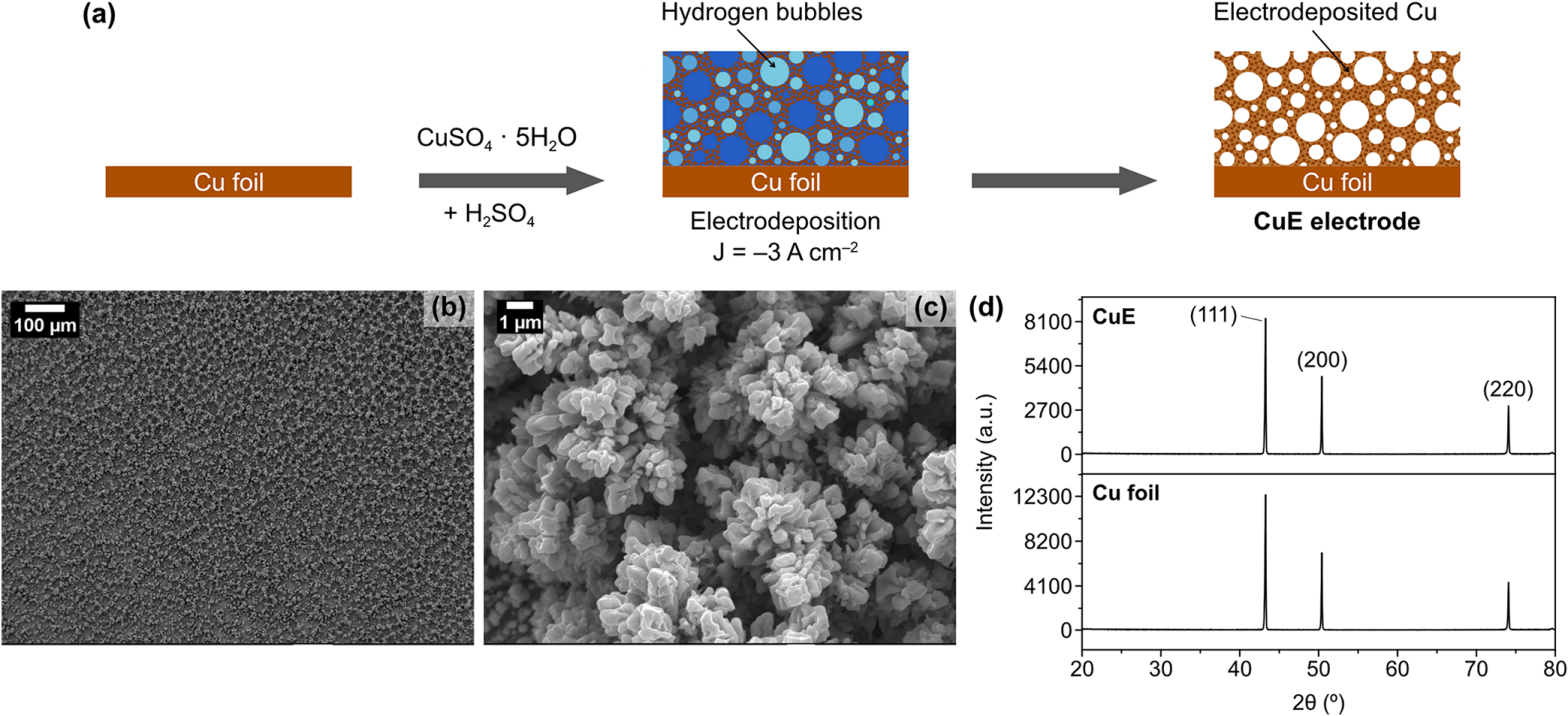
(a) Schematic illustration of CuE preparation via DHBT electrodeposition. (b, c) SEM images of CuE at low (100 μm) and high (1 μm) magnification. (d) XRD patterns of CuE (top) and Cu foil (bottom), showing characteristic diffraction peaks of face-centered cubic copper.

Scanning electron microscopy (SEM) images (Figure 2b–c) reveal a rough surface composed of copper dendrites interspersed with micron-scale pores, consistent with a substantially increased surface area. Electrochemical impedance spectroscopy (EIS) was used to estimate the electrochemically active surface area (ECSA) by extracting the double-layer capacitance (*C*_dl_) in a non-Faradaic potential region in KCl, KBr, and KI electrolytes. The electrochemical response was modeled using a modified Randles circuit (inset of Figure S1a) and compared to that of a smooth copper foil electrode. Nyquist plots acquired at −0.35 V versus the reversible hydrogen electrode (*V*_RHE_), a potential at which only capacitive processes associated with the double layer occur, are shown in Figure S1a.^40^ Analysis of the EIS data (Figure S1b–d) yields a *C*_dl_ corresponding to an effective surface area of 43.0 ± 1.0, 53 ± 3 and 24 ± 3 cm^2^ for CuE in KCl, KBr and KI, respectively, compared to 1 cm^2^ for the planar copper foil, confirming the substantially enhanced ECSA of the porous CuE electrode with the halide electrolytes. Although the measured ECSA varies with electrolyte identity, these differences do not directly correlate with catalytic performance, suggesting that electrolyte-specific interfacial interactions play a more dominant role than active surface area alone, as discussed in subsequent sections.

The X-ray diffraction (XRD) pattern of CuE (Figure 2d) exhibits well-defined diffraction peaks at 2θ ≈ 43°, 50°, and 74°, corresponding to the (111), (200), and (220) planes of the face-centered cubic copper (JCPDS 04–0836). The absence of additional reflections indicates that the electrodeposited layer retains the same crystalline phase as the copper foil substrate.

### 2.2 Reaction Pathways for Acetophenone Electrochemical Hydrogenation

Electrochemical hydrogenation of ACT to PE proceeds via two consecutive proton-coupled electron-transfer (PCET) steps with favorable energetics at room temperature.^17^ According to the mechanism proposed by Ciotti et al.^21^ in alkaline media, ACT is first hydrogenated at the carbonyl carbon, followed by hydrogenation at the oxygen atom to yield PE. In addition to this desired pathway, several competing reactions may occur under electrochemical conditions. For example, the initially formed ketyl radical intermediate (ACT^•^) can undergo radical–radical coupling with a second ACT^•^ species to yield the pinacol product, 2,3-diphenyl-2,3-butanediol.^41^ Moreover, further hydrogenation of PE may lead to ethylbenzene (ETB) via C–O bond hydrogenolysis.^42^ Together, these competing reaction pathways are summarized schematically in Figure 3. We note that, while prior mechanistic analyses have largely focused on alkaline media, hydrogenation under the near-neutral conditions employed in this work is likewise expected to proceed predominantly via surface-derived hydrogen species, rather than direct protonation by the aqueous electrolyte. Accordingly, hydrogenation is proposed to occur through hydrogen generated by water dissociation at the copper electrode, consistent with prior mechanistic proposals for ketone electrochemical hydrogenation.

**Figure 3:**
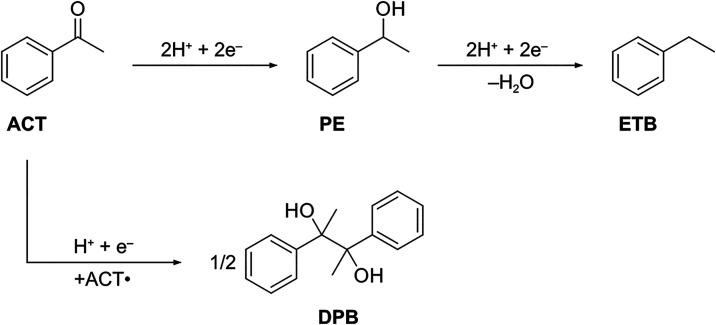
Proposed reaction pathways for the electrochemical hydrogenation of acetophenone (ACT) to 1-phenylethanol (PE), including competing dimerization to form the pinacol product 2,3-diphenyl-2,3-butanediol (DPB), and hydrogenolysis to ethylbenzene (ETB).

In aqueous media, ECH inevitably competes with the thermodynamically favored HER, which lowers the FE of the process. To delay HER without inducing base-promoted side reactions, we focus on mild, near-neutral conditions. Specifically, ACT electrochemical hydrogenation was investigated at pH ≈ 7 using a series of potassium electrolytes, including KCl, KBr, and KI, as well as a K_2_HPO_4_/KH_2_PO_4_ buffer system. These electrolytes provide stable reaction environments while enabling systematic evaluation of halide effects at the electrode–electrolyte interface.

Because of the limited aqueous solubility of ACT, a 9:1 water/ethanol (H_2_O/EtOH) cosolvent system was employed to enhance solubility without inducing salt precipitation. A control electrolysis experiment performed in the absence of ACT, combined with NMR analysis before and after reaction (Figure S2), shows no detectable changes in the ethanol signals, indicating that ethanol remains unreactive under the operating conditions. Thus, its role is primarily to improve substrate solubility and mass transport, consistent with previous reports under similar ECH conditions.^21,30,43^

To establish structure–reactivity relationships, we examined the effects of electrolyte composition, applied potential, halide identity, and ACT concentration on electrochemical hydrogenation performance. Chronoamperometry experiments were conducted in a two-compartment electrochemical cell separated by a cation-exchange membrane, with 15 mL of electrolyte solution in each compartment and a CuE geometric area of 1 cm^2^. All reactions were carried out for 4 h and repeated in triplicate to ensure reproducibility. Product identification and quantification were performed using high-performance liquid chromatography (HPLC); see Section S1 for further experimental details, and Table S1 and Figures S3–S4 for HPLC retention times, chromatograms, and calibration curves, respectively. Table S2 shows the lowest limit of concentration that can be detected (limit of detection, LOD) and the lowest level of concentration that can be quantified (limit of quantification, LOQ) obtained from the HPLC calibration method.

### 2.3 Electrolyte and Halide Effects on ECH Performance

The electrochemical response of CuE was first examined by linear sweep voltammetry (LSV) in 0.5 M KCl, KBr, KI, and KH_2_PO_4_/K_2_HPO_4_ electrolytes, both in the absence and presence of 25 mM ACT. All LSV measurements were recorded at a scan rate of 1 mV s^–1^ in a 9:1 H_2_O/EtOH mixture. Potentials are reported versus RHE, and current densities (*J*) were calculated by normalizing the measured currents to the exposed electrode geometrical area (1 cm^2^).

Figure 4 shows the LSVs obtained in 25 mM ACT in 0.5 M electrolyte concentration. In the absence of ACT, the cathodic current increases at more negative potentials due to the onset of the HER. The HER onset is operationally defined as the potential at which the current density reaches −2 mA·cm^–2^, a value selected to lie beyond the capacitive region and within a potential window where Faradaic contributions from HER can be clearly distinguished from acetophenone reduction. Although this definition is operational, the comparative trends between electrolytes are not sensitive to the exact current density threshold. In KCl electrolyte, this occurs at approximately −0.70 V_RHE_ (Figure 4a). When KH_2_PO_4_/K_2_HPO_4_ is used as the electrolyte, the HER onset shifts to significantly more positive potentials, occurring around – 0.30 V_RHE_ and accompanied by a steeper rise in cathodic current (Figure 4b). LSVs recorded in KBr and KI electrolytes (Figure 4c–d) show HER onset potentials comparable to those observed in KCl, with KBr exhibiting a slight positive shift, as highlighted in the inset of Figure 4c. Overall, comparison of the LSVs reveals a strong dependence of HER onset on electrolyte identity, with halide-containing electrolytes exhibiting substantially delayed HER relative to phosphate-buffered solutions, in agreement with previous reports by Choi and co-workers.^33^ Likewise, prior studies also demonstrate that halide adsorption on metal surfaces can modulate HER kinetics by altering hydrogen adsorption energetics and partially blocking active sites.^28,29^

**Figure 4:**
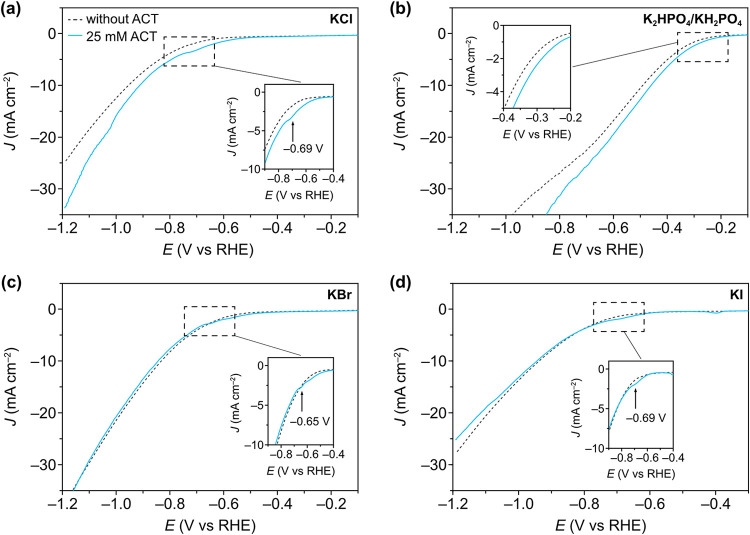
LSVs recorded with and without 25 mM ACT in 0.5 M (a) KCl, (b) KH_2_PO_4_/K_2_HPO_4_, (c) KBr, and (d) KI electrolytes. All measurements were performed in 9:1 H_2_O/EtOH mixture at a scan rate 1 mV s^–1^. The legend in panel (a) applies to all panels.

The addition of 25 mM ACT leads to pronounced and electrolyte-dependent changes in the LSV response. In KCl, introducing 25 mM ACT results in a positive shift of the apparent HER onset by approximately 0.10 V, accompanied by an increase in the slope of the *J*–*V* curve. These features indicate the emergence of an additional cathodic process that is attributed to ACT electrochemical hydrogenation. By contrast, adding ACT to KH_2_PO_4_/K_2_HPO_4_, KBr, or KI produces only a minor shift of approximately 0.02 V, with no appreciable change in slope. These trends suggest that, in the buffered phosphate electrolyte, proton reduction remains dominant and ACT reduction contributes only modestly to the overall cathodic current. Similarly, the comparable onset potential observed with and without ACT in KBr and KI indicate that ACT reduction can compete effectively with HER under these conditions, although with different efficiencies, as discussed below.

Consistent with these observations, a distinct cathodic feature appears at −0.69 V_RHE_ in KCl electrolyte, which is attributed to ACT electrochemical hydrogenation. A similar feature is also observed in KI at approximately the same potential, while in KBr it is shifted slightly to more positive potentials, appearing near −0.65 V_RHE_. In addition, increasing the ACT concentration in KCl produces a further positive shift in the apparent HER onset (Figure S5). This behavior suggests that ACT concentration contributes directly to the cathodic current, likely through adsorption-dependent interfacial effects. Such an interpretation is consistent with the established dependence of electrochemical hydrogenation rates and selectivity on substrate adsorption equilibria and surface hydrogen coverage, both of which modulate competition with HER and ultimately determine catalytic efficiency.^21,44^

Halide ions are known to induce surface restructuring of Cu electrodes under electrochemical conditions.^45^ Nevertheless, the comparable HER onset potentials observed in KCl, KBr, and KI electrolytes, together with the markedly different behavior in phosphate buffer, suggest that the trends reported here are governed predominantly by electrolyte-specific interfacial interactions rather than by extensive halide-induced surface reconstruction or etching.^33,46^ Furthermore, the mild near-neutral operating conditions employed in this work are not expected to promote significant morphological changes of the copper surface.^47^

To further assess the influence of the electrolyte composition on ACT electrochemical hydrogenation under steady-state conditions, KCl and a phosphate-buffered electrolyte (K_2_HPO_4_/KH_2_PO_4_) were directly compared. Both electrolytes were used at a concentration of 0.5 M and pH ≈ 7, and ECH reactions were carried out at – 0.69 V_RHE_ for 4 h. As shown in Figure 5a, the KCl electrolyte exhibits markedly superior performance. In KCl, ACT conversion reaches 76 ± 3% with a FE toward PE of 63.0 ± 1.0%. In contrast, when phosphate buffer is used, the conversion and FE decrease to 26 ± 12% and 2.5 ± 0.8%, respectively. Notably, the yield of PE reaches 72 ± 4% in KCl, whereas only 8.0 ± 2.0% is obtained in the phosphate-buffered electrolyte. These results demonstrate that KCl is significantly more effective than phosphate buffer for promoting selective ACT electrochemical hydrogenation under near-neutral conditions.

**Figure 5:**
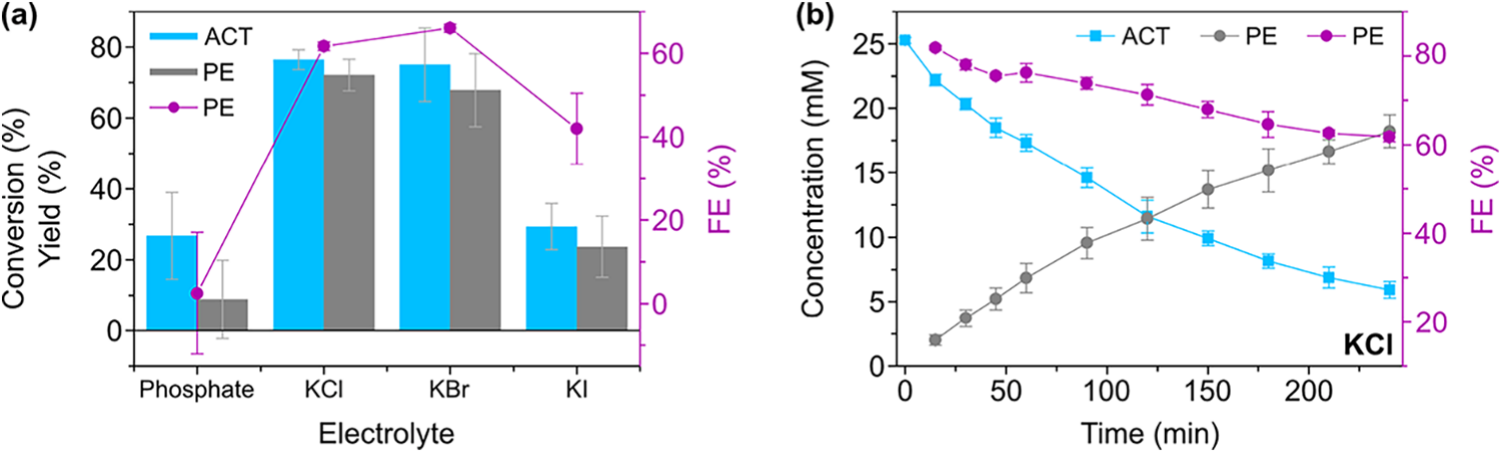
(a) Effect of electrolyte composition on the electrochemical hydrogenation of 25 mM ACT in 0.5 M electrolytes at pH ≈ 7 (electrolyte compositions include KH_2_PO_4_/K_2_HPO_4_, KCl, KBr, and KI). ACT conversion and PE yield are shown on the primary axis, while FE toward PE is shown on the secondary axis. (b) Time-resolved concentration profiles for the electrochemical hydrogenation of 25 mM ACT in 0.5 M KCl. All chronoamperometry experiments were performed for 4 h at −0.69 V_RHE_ in a 9:1 H_2_O/EtOH mixture.

Time-resolved concentration profiles further illustrate these differences in selectivity. In KCl, the FE toward PE begins at 81.8 ± 0.5% and remains relatively stable in the range of 70 to 80% over most of the reaction duration, before decreasing to 63.0 ± 1.0% after 4 h (Figure 5b). In contrast, the phosphate electrolyte exhibits a pronounced loss of selectivity over time, with the FE decreasing to approximately 2% after 4 h (Figure 5a).

The superior performance observed in KCl is attributed to its ability to delay HER while enabling selective ACT electrochemical hydrogenation. This interpretation is supported by the LSV data (Figure 4a–b). In the absence of ACT, cathodic currents associated with HER are significantly lower in KCl than in phosphate electrolyte, indicating suppressed proton reduction. Upon addition of ACT, KCl exhibits a modest increase in current that can be attributed to selective organic reduction. In phosphate electrolyte, by contrast, the elevated HER background current obscures any distinct contribution from ACT reduction. This difference can be rationalized by considering the role of the electrolyte species at near-neutral pH. Phosphate ions can act as proton donors and facilitate HER by supplying hydrogen to the electrode surface.^48–50^ In contrast, KCl and the other halide electrolytes do not act as proton donors; therefore water remains the sole proton source, which limits hydrogen availability for HER and favors ECH selectivity.^30,33^ This behavior is consistent with prior reports of CO_2_ electroreduction, particularly in KCl electrolytes, which show that chloride ions interact specifically with copper surfaces, either by delaying HER or by stabilizing adsorbed reaction intermediates.^29,46,51^

Additionally, contact angle measurements were performed using all investigated electrolytes on the porous CuE surface. In all cases, the droplets spread rapidly across the electrode showing high hydrophilic behavior (Figure S6), preventing reliable determination of equilibrium contact angles. This behavior is consistent with the strong wetting commonly observed for rough, porous metallic electrodes prepared by electrodeposition methods.^52,53^ In such systems, surface roughness and capillary infiltration amplify wetting, often leading to near-complete liquid uptake and making precise equilibrium contact angle measurements challenging.^54^ Although these measurements do not allow quantitative comparison of equilibrium wettability among electrolytes, they show no qualitative evidence for pronounced electrolyte-dependent wetting differences. Therefore, the observed differences in HER suppression and ECH performance are more likely governed by electrolyte-specific interfacial chemistry than by macroscopic wettability effects.

Having established the role of halide-containing electrolytes in promoting selective ACT electrochemical hydrogenation, next we examine how the specific halide species (Cl^–^, Br^–^, or I^–^) influence reaction kinetics, FE, and product selectivity under identical operating conditions.

To isolate the effect of each halide anion, ACT electrochemical hydrogenation was compared in KCl, KBr, and KI electrolytes, each at a concentration of 0.5 M, at −0.69 V_RHE_ for 4 h. As shown in Figure 5a, halide identity strongly influences both FE and product selectivity. KCl and KBr yield comparable performance, with ACT conversions of 76 ± 3% and 75 ± 10%, and FEs of 63.0 ± 1.0% and 65.0 ± 1.0%, respectively. In contrast, KI exhibits substantially lower activity and selectivity, with 29 ± 6% conversion, and a FE of 42 ± 8%. Correspondingly, the yield of PE reaches 72 ± 4% in KCl and 68 ± 10% in KBr, whereas only 24 ± 8% is obtained in KI. Furthermore, the carbon balance based on HPLC quantification for the different halide electrolytes was within 95% range (Table S3).

Time-resolved kinetic profiles (Figures 5b and S7) further highlight these differences. Reactions conducted in KCl and KBr approach near-complete conversion within 4 h, whereas KI displays markedly slower kinetics and incomplete ECH over the same time period. In addition, the FE in KI decreases progressively with time, indicating less efficient charge utilization toward PE formation. Chronoamperometric measurements show relatively stable current densities throughout electrolysis (Figure S8), supporting the reliability of the FE values and indicating that the observed trends do not arise from significant electrode deactivation.

To evaluate whether the catalytic trends originate from differences in active surface area, the ECSA values determined in Section 2.1 were compared with the electrochemical performance. Although variations in ECSA are observed among the halide electrolytes, no direct correlation with catalytic activity is evident. For example, KBr exhibits the largest ECSA but does not proportionally outperform KCl in either activity or selectivity toward PE formation. These observations indicate that electrolyte-dependent catalytic behavior is governed primarily by specific interfacial interactions between halide anions and the Cu surface rather than by active surface area effects alone.

Furthermore, although CuE exhibits a substantially larger ECSA than planar Cu foil, control experiments performed using Cu foil under identical conditions show negligible ACT conversion within the detection limits of HPLC (Figure S9). This result indicates that the enhanced catalytic performance of CuE arises not only from increased surface area, but also from the porous morphology and its influence on substrate accessibility, local interfacial environments, and charge-transfer processes. As discussed in subsequent sections, this interpretation is further supported by complementary kinetic, spectroscopic, and computational evidence.

These trends are consistent with differences in halide–copper interactions. While our previous work emphasized the importance of competitive adsorption between water and ACT under alkaline conditions,^21^ the present results demonstrate that the halide anion provides an independent means of tuning surface coverage, reaction kinetics, and selectivity under near-neutral conditions. Consistent with this interpretation, prior studies have also shown that Br^–^ and I^–^ adsorb more strongly on Cu surfaces than Cl^–^,^55–57^ a behavior that is reflected in the halide-dependent HER suppression observed in the LSVs (Figure 4). In the present system, Cl^–^ and Br^–^ provide a favorable balance between delaying HER and maintaining sufficient surface accessibility for ACT adsorption and charge transfer, thereby promoting efficient electrochemical hydrogenation. In contrast, the stronger adsorption of I^–^ limits the number of surface sites available for ACT adsorption.^46,51,58^ Because ACT is proposed to adsorb through its π-conjugated system during ECH (*vide infra*), partial halide desorption is required for efficient hydrogenation to occur. In addition, persistent adsorption of iodide under moderately cathodic conditions may further alter interfacial charge transfer and restrict surface accessibility,^59^ thereby contributing to the lower FE observed in KI electrolyte. This interpretation is consistent with the DFT calculations discussed in Section 2.7, which indicate that iodide remains adsorbed on Cu over a broader cathodic potential range than chloride or bromide, effectively blocking active sites required for ACT electrochemical hydrogenation.

### 2.4 Effect of Applied Potential on ECH Performance

To examine the influence of applied potential on the ECH of ACT, additional control experiments were conducted at four cathodic potentials (i.e., −0.59, −0.69, −0.79, and −0.89 V_RHE_) for 4 h using an ACT concentration of 25 mM. These potentials were selected based on the reduction feature observed in the LSVs (Figure 4a) and were chosen to probe the balance between ACT hydrogenation and the competing HER. Although KCl and KBr exhibited comparable performance in earlier experiments, 0.5 M KCl was selected as the electrolyte for all subsequent studies to ensure consistency with prior sections and earlier literature.

At −0.59 V_RHE_, the reaction reaches an ACT conversion of 39 ± 3% and a PE yield of 33 ± 4% after 4 h (Figure 6a). The corresponding kinetic profile (Figure S10a) indicates that the reaction does not reach completion within this time window. Under these conditions, the FE initially approaches 70% and gradually decreases to 53.0 ± 1.0% over the course of the experiment, suggesting that ACT reduction becomes increasingly limited as the reaction proceeds.

**Figure 6:**
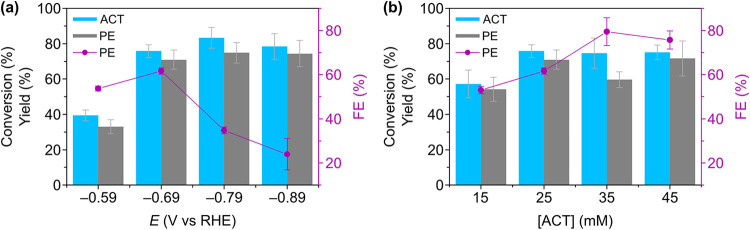
(a) Effect of applied potential on the electrochemical hydrogenation of 25 mM ACT in 0.5 M KCl. (b) Effect of ACT concentration (15, 25, 35, and 45 mM) on electrochemical hydrogenation in 0.5 M KCl at −0.69 V_RHE_. All chronoamperometry experiments were performed for 4 h in a 9:1 H_2_O/EtOH solvent mixture.

As the applied potential becomes more cathodic, both conversion and yield increase, and the reaction progresses more rapidly toward completion. Optimal performance is observed at −0.69 V_RHE_, where ACT conversion and PE yield reach 76 ± 3% and 72 ± 4%, respectively, with an FE of 63.0 ± 1.0% after 4 h (Figure 6a). The time-resolved concentration profile at this potential (Figure 5b) shows that the FE remains close to 80% during the first hour of electrolysis. Over this period, the ACT concentration decreases steadily while the PE concentration increases linearly, consistent with selective ECH to the alcohol. At longer reaction times, the FE gradually decreases to values around 60–70%, which we attribute to restricted ACT mass transport and local substrate depletion near the electrode surface, conditions under which HER becomes comparatively more competitive.

At more negative potentials (−0.79 and −0.89 V_RHE_), ACT conversion and PE yield remain high (Figure 6a); however, the FE decreases substantially, reaching 35.0 ± 1.0% at −0.79 V_RHE_ and 24 ± 7% at −0.89 V_RHE_. This trend reflects the increasing contribution of HER at more cathodic potentials, as also evidenced by the corresponding kinetic profiles (Figure S10b,c). Notably, no pinacol coupling product is detected across the entire potential range investigated. The chronoamperometric curves recorded at each applied potential show stable current densities throughout electrolysis (Figure S11), indicating that the observed differences in FE are not due to pronounced electrode instability. In addition, carbon balance values remain between 92 and 97% across the investigated potentials (Table S4), indicating that no major liquid products remain undetected within the analytical limits of the HPLC method (<0.001 mM; Table S2).

To further assess product selectivity, an additional control experiment was performed using 25 mM PE under standard reaction conditions (0.5 M KCl, 9:1 H_2_O/EtOH) at −0.69 V_RHE_ for 4 h. No formation of the hydrogenolysis product ETB is observed (Figure S12), confirming the high selectivity of the system toward PE formation.

Taken together, these results highlight the critical role of applied potential in tuning reaction rate, Faradaic efficiency, and product selectivity during ACT electrochemical hydrogenation. On this basis, −0.69 V_RHE_ is identified as the optimal operating potential, providing the best compromise between high conversion, high yield, and efficient charge utilization while maintaining exclusive selectivity toward PE.

### 2.5 Effect of Acetophenone Concentration on ECH Performance

Having established the influence of the applied potential, we next examined the effect of increasing ACT concentration (15, 25, 35, and 45 mM) under the optimized reaction conditions, namely 0.5 M KCl in a 9:1 H_2_O/EtOH solvent mixture at −0.69 V_RHE_ for 4 h.

As shown in Figure 6b, increasing the ACT concentration leads to higher conversion and PE yield, accompanied by an improvement in FE, which reaches a maximum of 80 ± 6% at 35 mM ACT. Although the FE is highest at 35 mM, the value obtained at 45 mM remains comparable within experimental error (76 ± 4%). Importantly, increasing the ACT concentration from 35 to 45 mM enhances the PE yield from 62 ± 4% to 72 ± 9%, while the conversion remains essentially unchanged. This behavior is attributed to increased ACT surface coverage at higher bulk concentrations, which improves reactant availability at the electrode–electrolyte interface.

Time-resolved reaction profiles further reinforce this trend (Figure S13). For ACT concentrations of 35 and 45 mM, the FE reaches values as high as 90% at early reaction times and remains near this level for the first 1.5–2 h of electrolysis. At longer reaction times, the FE gradually decreases to approximately 80% as the reaction proceeds. This behavior is consistent with partial ACT depletion near the electrode surface and increasing mass-transport limitations between the bulk electrolyte and the interface. The chronoamperometry curves recorded for all tested ACT concentrations show relatively stable current densities throughout electrolysis (Figure S14), supporting consistent electrode operation over the reaction time.

To further confirm HER as a competing process under the optimized conditions (45 mM ACT in 0.5 M KCl at −0.69 V_RHE_), hydrogen evolution was monitored using microgas chromatography by directly coupling the electrochemical cell to the instrument. As shown in Figure S15, the H_2_ signal increases substantially toward the end of the reaction, consistent with enhanced HER activity. Quantitative analysis using the calibration curve in Figure S16 yields an H_2_ FE of 20% after 4 h of electrolysis. Combined with the FE toward PE of 76 ± 4%, the total quantified FE reaches approximately 96% (Figure S17), indicating that the dominant cathodic processes are accounted for under these conditions. Consistent with this result, the carbon balance under the optimized conditions is 97 ± 4% (Table S4), supporting the absence of significant unquantified liquid-phase products within the sensitivity of the HPLC method (Table S2).

Notably, across the entire concentration range investigated, the system remains highly selective toward the alcohol product, with no evidence of pinacol coupling or hydrogenolysis products. HPLC chromatograms recorded before and after electrolysis show no additional signals attributable to these side reactions within the detection limits of the method (Figure S18). These results further highlight the beneficial role of halide-containing electrolytes in promoting efficient and selective ECH, particularly under conditions of increased substrate availability at the electrode surface.

### 2.6 Mechanistic Insights from Kinetic Studies and In Situ Spectroscopy

The dependence of ECH on applied potential and ACT concentration provides a basis for probing the underlying reaction mechanism. To further clarify the reaction pathway for ACT electrochemical hydrogenation on CuE, we examined how the reaction rate depends on ACT concentration and directly probed surface adsorption phenomena using electrochemical in situ surface-enhanced Raman spectroscopy (EC-SERS).

In a Langmuir–Hinshelwood mechanism, two surface-bound species, such as adsorbed ACT and surface hydrogen, react directly on the electrode surface.^60,61^ In contrast, an Eley–Rideal mechanism involves a surface-adsorbed organic species reacting directly with a solution-phase proton.^37^ Schematic representations of both mechanisms are shown in Figure S19.

Following the kinetic analysis reported by Duan and co-workers,^15^ the apparent reaction order was determined from kinetic experiments performed at ACT concentrations of 15, 25, 35, and 45 mM (Figure S20a and Table S5). A log–log analysis of the initial reaction rate as a function of ACT concentration yields an apparent reaction order of 0.80 ± 0.20 (Figure S20b). This subunity dependence indicates that the reaction rate does not scale proportionally with the bulk ACT concentration and instead reflects negative coverage-dependent effects, such as adsorption saturation, competition between solvent and substrate for surface sites, or possible mass transport limitations.^62^ Although this observation alone does not allow discrimination between L–H and E–R pathways, it indicates that interfacial surface availability and adsorbate coverage play a significant role under the conditions studied.

We note that Choi and co-workers^16,37^ proposed that E–R pathways are favored in acidic media, where high proton availability near the electrode enables direct hydrogenation of adsorbed molecules. Under the near-neutral conditions employed here, the limited proton availability may disfavor a purely E–R-type pathway and instead favor pathways involving surface-bound hydrogen species. To further probe the role of surface-bound hydrogen species, we performed a quenching experiment using 2,4-dichlorophenol (2,4-DCP). This compound is known to accumulate on metal electrode surfaces and selectively scavenge adsorbed hydrogen atoms through hydrodehalogenation.^63,64^

As shown in Figure 7a, the presence of 2,4-DCP substantially reduces reaction performance. In the absence of 2,4-DCP, the reaction reaches 76 ± 3% ACT conversion and 63.0 ± 1.0% FE. Upon addition of 2,4-DCP, these values decrease to 33 ± 7% conversion and 48 ± 7% FE under otherwise identical conditions (−0.69 V_RHE_, 25 mM ACT, 4 h). This decrease supports the participation of surface-adsorbed hydrogen species in the ECH process and is consistent with a surface-mediated hydrogenation pathway, although additional interfacial effects arising from 2,4-DCP adsorption cannot be fully excluded. The corresponding time-resolved concentration profile for the quenching experiment is shown in Figure S21.

**Figure 7:**
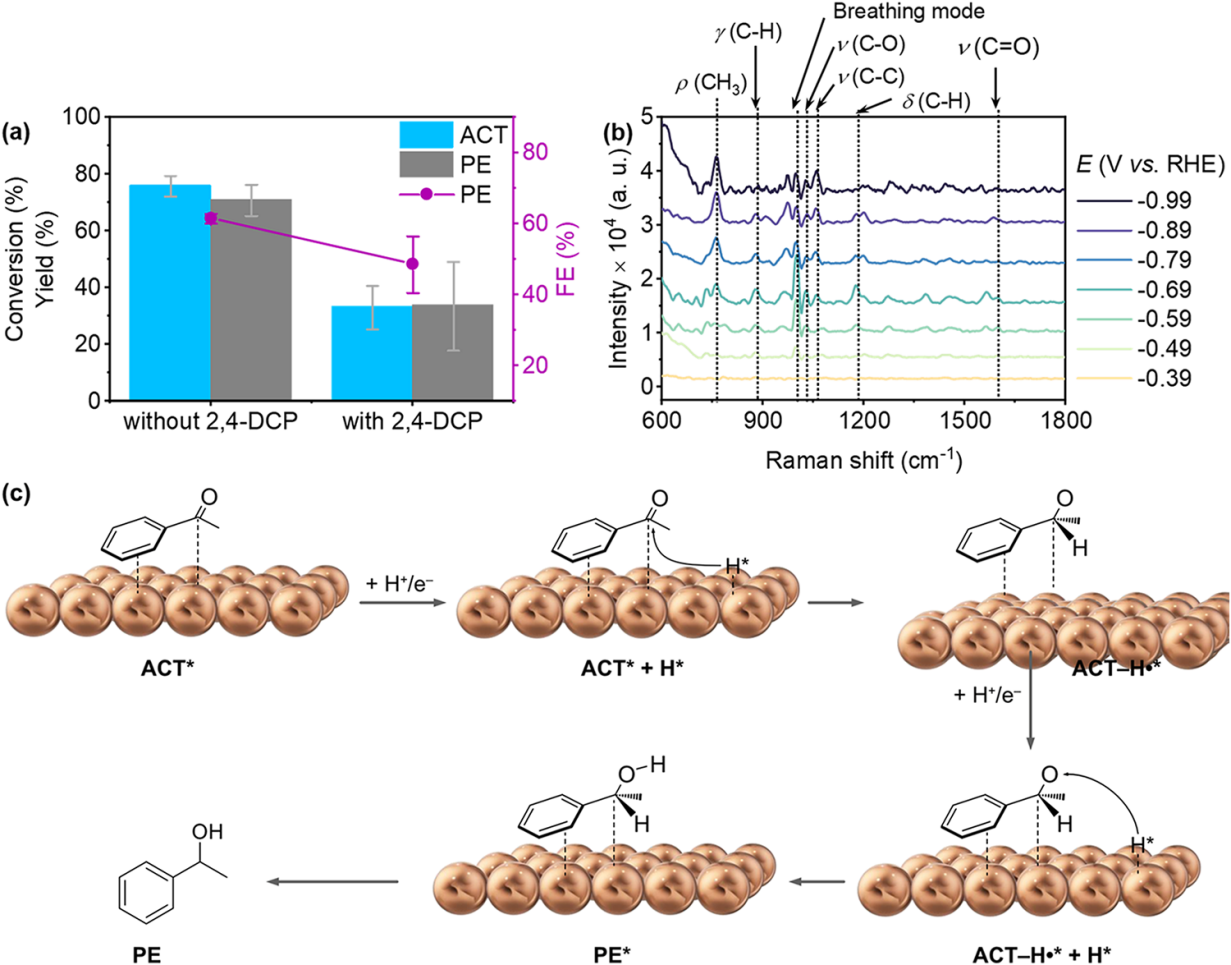
(a) Effect of 25 mM 2,4-DCP as a quenching agent for the ECH of 25 mM ACT in 0.5 M KCl at −0.69 V_RHE_ for 4 h, showing a decrease in performance metrics with the introduction of 2,4-DCP. (b) In situ EC-SERS for the ECH of 45 mM ACT in 0.5 M KCl (ν: stretching; ρ: rocking; γ: out-of-plane bending; δ: in-plane bending). (c) Schematic illustration of the proposed L–H pathway for the ECH of ACT to PE.

Additional mechanistic insight was obtained from in situ EC-SERS, which was used to identify adsorbed species under reaction conditions (see Section S1.5.5 and Figure S22 for experimental details). As shown in Figure 7b, a Raman band at 999 cm^–1^, assigned to the ring-breathing mode of ACT,^65,66^ becomes spectroscopically detectable at potentials more negative than −0.39 V_RHE_. The emergence of this signal supports a planar adsorption geometry of ACT on the copper surface, consistent with prior experimental observations by Bondue and Koper^66^ and with π-system-directed adsorption predicted by theoretical studies.^21^ We note that this ring-breathing mode may also be present at more positive potentials, but with reduced intensity due to lower surface coverage, less favorable molecular orientation, or rapid exchange with solution.^67^ Additional bands observed at approximately 890 cm^–1^ and 1060 cm^–1^ are assigned to γ­(C–H) out-of-plane bending and ν­(C–C) aromatic ring stretching modes, respectively, in agreement with literature reports for monosubstituted benzene derivatives.^68,69^ We note that the overall signal-to-noise ratio is limited under operando conditions because of the experimental configuration and the inherently weak Raman scattering of adsorbed species. Therefore, only well-defined and reproducible spectral features are considered in the analysis. A weak feature centered at 1597 cm^–1^ may be assigned to ν­(C=O) symmetric stretching; however, because this band approaches the noise level, this assignment should be interpreted with caution.^65^ The full potential-dependent Raman spectra are shown in Figure S22.

At more negative potentials (below −0.69 V_RHE_), additional spectral features emerge that may correspond to reaction intermediates. For example, a band at 760 cm^–1^ is consistent with CH_3_ rocking ρ­(CH_3_) vibrations, while a feature at 1030 cm^–1^ can be assigned to C–O stretching, suggesting the presence of an alkoxy-type intermediate.^25,70,71^ Moreover, a band at 1178 cm^–1^ appears under strongly reducing conditions and may arise from δ­(C–H) in-plane bending coupled with aromatic ring modes associated with surface-bound intermediate species.^27^ The attenuation of the ring-breathing mode and carbonyl-related features at more negative potentials suggests changes in the adsorption geometry and/or electronic structure of the aromatic system, possibly associated with the formation of surface-bound reaction intermediates, as previously reported.^72,73^ Although Raman reference spectra for these intermediates are limited, these assignments are supported by spectral features of structurally related compounds and prior reports on ACT electrochemical hydrogenation.^21^

Overall, the kinetic analysis, quenching experiments, and EC-SERS measurements support a surface-mediated hydrogenation pathway consistent with an L–H-like mechanism for ACT electrochemical hydrogenation on CuE (Figure 7c), while not definitively excluding contributions from alternative pathways. In this pathway, ACT adsorbs through its aromatic π-system, while surface-derived hydrogen species are generated via water dissociation. Adsorbed hydrogen then reacts with the carbonyl carbon to form a surface-bound radical intermediate (ACT–H•), followed by a second PCET step at the oxygen atom to yield PE as the final product.

### 2.7 Halide-Dependent Surface Coverages under ECH Conditions: Insights from DFT Calculations

Periodic DFT calculations (see Section S8 for computational details) were performed to rationalize the experimentally observed electrolyte-dependent trends in ACT electrochemical hydrogenation. The copper electrode was modeled as a flat Cu(111) surface, and surface coverage analyses were carried out to evaluate the relative stability of hydrogen and halide adsorbates under electrochemically relevant conditions. As discussed below, these calculations indicate that differences in electrolyte performance arise from the interplay between hydrogen adsorption and halide surface coverage, or the absence thereof (Figure 8).

**Figure 8:**
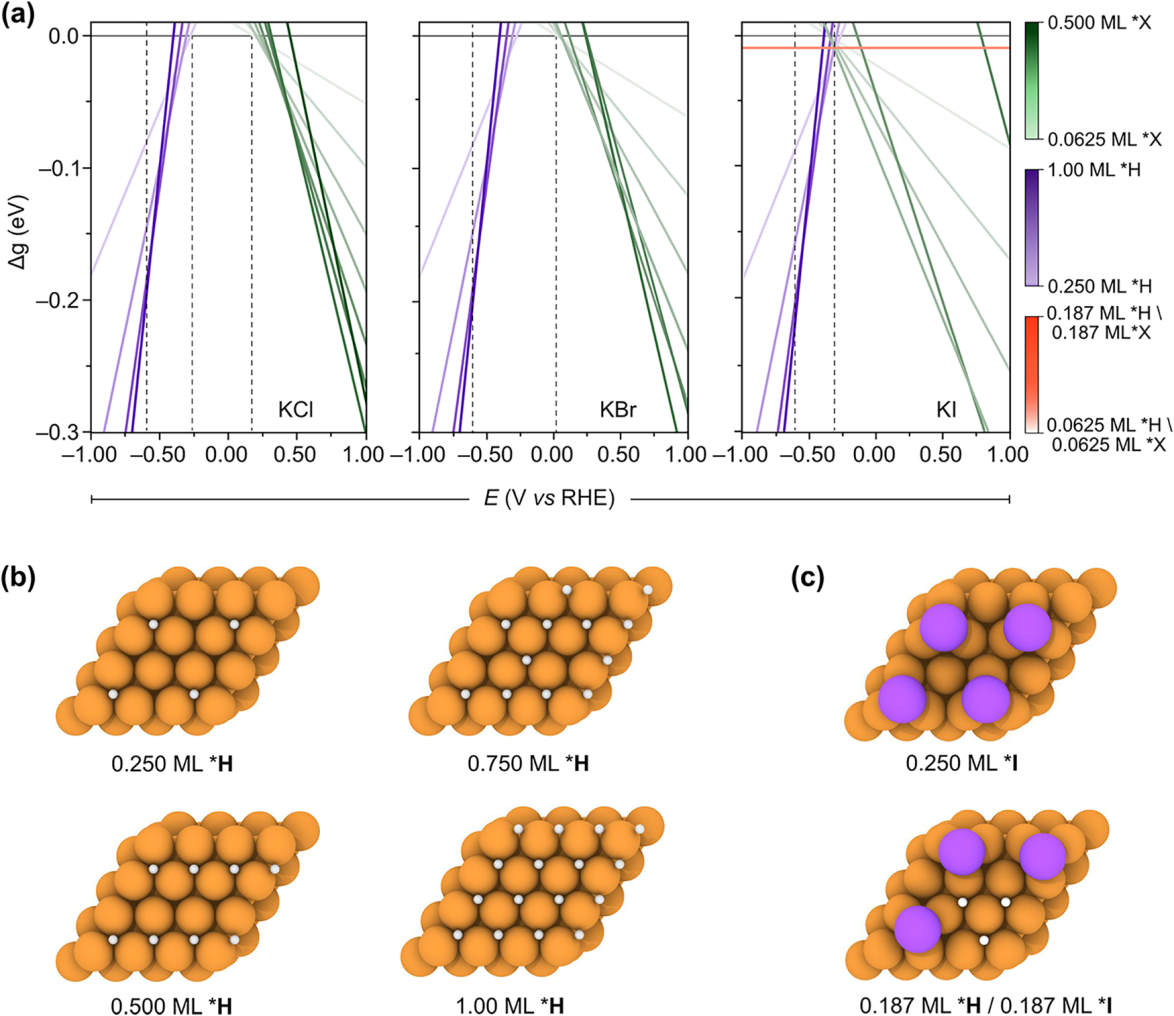
(a) Calculated surface coverage maps relative to the bare Cu(111) surface (gray solid line) for hydrogen and halide adsorption, shown for chloride (left), bromide (middle), and iodide (right) as a function of applied potential. Color bars denote surface coverages of the halide (*X, green), hydrogen (purple), and mixed *H–*X configurations (red). (b) Predicted Cu(111) resting states under increasing hydrogen coverage, up to 1.00 monolayer (ML) *H. (c) Predicted Cu(111) resting states in KI electrolyte at *ca*. −0.30 V_RHE_, showing 0.250 ML *I coverage (top) and a mixed coverage of 0.187 ML *H and 0.187 ML *I (bottom). All optimized structures are shown in Figures S23–S25. The adsorption Gibbs energies, Δ*g*, are normalized by the number of surface Cu atoms, as described in Section S8.

As shown in Figure 8a, the Cu(111) surface in the presence of KCl and KBr is predicted to be effectively halide-free under open-circuit conditions, remaining largely bare until hydrogen adsorption on *fcc* sites becomes thermodynamically favorable at potentials more negative than −0.25 V_RHE_. The relative stability of halide adsorption varies gradually across the halide series. Sparse bromide coverages remain marginally stable near 0.00 V_RHE_, whereas chloride desorbs at slightly more positive potentials, around +0.20 V_RHE_. Under increasingly cathodic conditions, however, both chloride and bromide are rapidly depleted from the surface. This behavior is consistent with experimental observations showing that these electrolytes do not hinder ACT electrochemical hydrogenation.

In contrast, iodide exhibits a markedly different behavior. As illustrated in Figure 8c, iodide coverages of 0.250 monolayers (ML) remain thermodynamically stable at potentials below −0.25 V_RHE_. Interestingly, lower iodide coverages are comparatively less stable than those of chloride and bromide even under oxidizing conditions, suggesting that iodide adsorption benefits from lateral stabilization at moderate surface densities. At higher coverages, however, iodide adsorption becomes unfavorable due to steric constraints and ultimately leads to I_2_ evolution. This behavior contrasts with chloride and bromide, which can accommodate higher surface coverages owing to their smaller ionic radii and reduced steric hindrance.

The persistence of stable iodide coverages under cathodic conditions provides a molecular-level explanation for the reduced ECH activity observed experimentally in KI electrolytes. By occupying surface sites required for ACT adsorption, iodide effectively limits access of the organic substrate to the copper surface, thereby suppressing ECH despite the delayed onset of HER.

Beyond halide adsorption, DFT calculations also provide insight into hydrogen site occupancy. Regardless of the halide considered, the thermodynamically favored resting state of the Cu(111) surface at markedly cathodic potentials (below −0.5 V_RHE_) corresponds to a full monolayer of hydrogen (1.00 ML *H, Figure 8b). For chloride- and bromide-containing electrolytes, no stable halide coadsorption with hydrogen is predicted across the operating potential window, as hydrogen progressively occupies available *fcc* sites on an otherwise bare surface.

In contrast, for KI electrolytes at intermediate cathodic potentials (ca. −0.30 V_RHE_), our calculations predict the possible coexistence of iodide and hydrogen adsorbates on the Cu(111) surface. Several mixed *H–*I configurations, including coverages of 0.187 ML *H and 0.187 ML *I (solid red line in Figure 8c), are found to be nearly isoenergetic with the corresponding single-adsorbate resting states. Although such mixed coverages are expected to vanish at more negative potentials as hydrogen adsorption becomes dominant, their relative stability underscores the greater persistence of iodide on the copper surface compared to chloride and bromide.

Importantly, even in the presence of residual iodide, hydrogen adsorption remains thermodynamically favorable at multiple surface *fcc* sites. As a result, HER can still proceed efficiently in KI electrolytes while ACT electrochemical hydrogenation is simultaneously suppressed due to limited substrate adsorption. Conversely, the absence of stable halide coverages in KCl and KBr at working potentials leaves surface sites accessible for ACT adsorption, providing a consistent explanation for their superior ECH performance relative to KI.

## 3 Conclusions

In this work, we investigated the electrochemical hydrogenation of acetophenone to 1-phenylethanol on copper-based electrodes as a model system for the valorization of biomass-derived ketones. Copper was selected because of its moderate intrinsic activity toward the hydrogen evolution reaction (HER), ease of preparation, and favorable interactions with aromatic organic substrates.

In contrast to conventional strategies that rely on strongly alkaline electrolytes to suppress HER, we demonstrate that neutral halide electrolytes, specifically KCl and KBr, enable efficient and selective electrochemical hydrogenation under mild conditions. Systematic optimization of key reaction parameters, including applied potential, electrolyte identity, and acetophenone concentration, identifies – 0.69 V_RHE_ and 45 mM acetophenone in 0.5 M KCl (9:1 H_2_O:EtOH) as optimal conditions, where increased substrate concentration provides a favorable balance between high Faradaic efficiency and enhanced product yield.

Building on these performance trends, mechanistic investigations combining kinetic analysis, hydrogen scavenging experiments, and electrochemical in situ surface-enhanced Raman spectroscopy suggest a surface-mediated hydrogenation pathway on copper under neutral conditions. Collectively, these results are consistent with a Langmuir–Hinshelwood-like mechanism involving adsorbed acetophenone and surface-derived hydrogen species generated through water dissociation, although contributions from alternative pathways cannot be fully excluded. While related surface-mediated pathways have previously been discussed under alkaline conditions, the present work demonstrates that similar interfacial chemistry can be achieved through electrolyte engineering at neutral pH, without reliance on pH-driven HER suppression.

Within this framework, electrolyte identity emerges as a decisive factor governing reaction performance. Compared to phosphate-buffered electrolytes, KCl and KBr delay the onset of HER and enable effective charge transfer toward organic reduction. Among the halides examined, KCl and KBr outperform KI, which exhibits lower activity and faradaic efficiency. This behavior is attributed to the strong and persistent adsorption of iodide on the copper surface at moderately cathodic potentials, which limits acetophenone adsorption despite the delayed HER onset.

At the atomic level, density functional theory calculations provide a molecular-level explanation for these trends. Specifically, the calculations show that chloride and bromide desorb readily under reaction conditions, leaving surface sites accessible for acetophenone adsorption, whereas iodide remains stable and can coexist with hydrogen on the surface over a broad potential range. Together with the experimental results, these findings highlight halide identity as a powerful and tunable lever for controlling surface coverage, reaction kinetics, and selectivity during electrochemical hydrogenation.

Overall, this work demonstrates that electrolyte engineering, and specifically the judicious choice of halide anions, enables efficient and selective electrochemical hydrogenation of ketones under mild, near-neutral conditions. These findings set the groundwork for the rational design of high-performance electrocatalytic systems for biomass upgrading and other selective hydrogenation reactions.

## Supplementary Material



## Data Availability

Computational data underlying this work, including Cartesian coordinates and energies of the optimized structures are openly accessible in the following ioChem-BD dataset: https://doi.org/10.19061/iochem-bd-6-642 All experimental data is available on repository: (10.5281/zenodo.20640717).

## Notes

Experimental procedures, estimation of the electrochemically active surface area of CuE electrodes, control experiments for cosolvent stability, product separation and quantification by HPLC, acetophenone electrochemical hydrogenation optimization and corresponding kinetic analysis, electrochemical in situ surface-enhanced Raman spectroscopy results, and computational methods (PDF)
