# Supplementary material for: Halide Electrolyte Effects in the Electrochemical Hydrogenation of Ketones on Copper
Source: JACS Au. 2026 Jun 23;6(7):3889–902. doi: 10.1021/jacsau.6c00452 (PMC13417248; doi:10.1021/jacsau.6c00452)
Supplement: Supplementary file 1 [file au6c00452_si_001.pdf]

Supporting Information

Halide Electrolyte Effects in the Electrochemical Hydrogenation of Ketones on Copper

Jose Solera-Rojas,[1] Didac A. Fenoll,[2] Elena Segura-Sanchis,[1] M. Consuelo Barrantes,[1] Conor Brennan-Pollak,[3] Francisco Fabregat-Santiago,[1] Carmen Mejuto,[1*] Max García-Melchor,[2,3,4*] and Elena Mas-Marzá[1*]

[1]*Institute of Advanced Materials (INAM), Universitat Jaume I, 12006 Castelló, Spain.*
[2]*CIC energiGUNE, Parque Tecnológico de Álava, C/ Albert Einstein 48, 01510 Vitoria-Gasteiz, Spain.*
[3]*School of Chemistry, Trinity College Dublin, College Green 2, Dublin 2, Ireland.*
[4]*IKERBASQUE, Basque Foundation for Science, Plaza de Euskadi 5, 48009 Bilbao, Spain.*

*Corresponding authors: mejuto@uji.es; maxgarcia@cicenergigune.com; emas@uji.es

Table of Contents

S1. Experimental Section **S3**

S1.1. Materials **S3**

S1.2. Copper Electrodes Preparation **S3**

S1.3. Electrochemical Measurements **S4**

S1.3.1. Impedance Spectroscopy and Active Surface S4

S1.3.2. Linear Sweep Voltammetry S5

S1.3.3. Chronoamperometry Experiments S5

S1.4. Nafion™ 117 Membrane Activation **S6**

S1.5. Characterization Techniques **S6**

S1.5.1. HPLC Quantification S6

S1.5.2. Scanning Electron Microscopy S7

S1.5.3. X-Ray Diffraction Pattern S7

S1.5.4. Micro Gas Chromatography S7
S1.5.5. Electrochemical in situ Surface-Enhanced Raman Spectroscopy (EC-SERS) S7
S1.5.6. Nuclear Magnetic Resonance Spectroscopy (NMR) S8
S1.5.7. Contact Angle Measurements S8

S2. Estimation of the Electrochemical Active Surface Area of CuE Electrodes..... S9

S3. Control Experiments for the Co-solvent Stability S10

S4. Product Separation and Quantification by HPLC S11

S5. Acetophenone Electrochemical Hydrogenation Reaction Optimization S14

S6. Kinetic Analysis for the ECH of ACT S25

S7. Electrochemical in situ Surface-Enhanced Raman Spectroscopy Results...... S28

S8. Computational Methods S29

S9. References S35

S1. Experimental Section

S1.1. Materials

All chemical reagents were obtained from commercial sources and used without further purification. All solutions were prepared using ultrapure water (Milli-Q® gradient, ≥18.2 MΩ cm), unless otherwise stated.

The materials used include Cu foil (99.9%, 0.2 mm thickness, GoodFellow, Inc.), $CuSO_4 \cdot 5H_2O$ (99.999% trace metal basis, Sigma-Aldrich), acetophenone (99%, Thermo Scientific), DL-*sec*-phenethyl alcohol (97%, Thermo Scientific), ethylbenzene (for synthesis, Sigma-Aldrich), 2,3-diphenylbutane-2,3-diol (>98.0%, TCI), H_2SO_4 (ACS reagent, 95.0-98.0%, Sigma-Aldrich), HCl (laboratory reagent grade, ~97%, Fisher Scientific), HNO_3 (laboratory reagent grade, ~70%, Fisher Scientific), H_2O_2 solution (30% w/w, puriss, Sigma-Aldrich), ethanol (SupraSolv® for gas chromatography ECD and FID, Sigma-Aldrich), KCl (99.999% trace metal basis, Thermo Scientific), Na_2SO_3 (98%, anhydrous, Thermo Scientific), KBr (FT-IR grade, ≥99% trace metals, Sigma-Aldrich), KI (BioXtra, ≥99.0%, Sigma-Aldrich), KH_2PO_4 (ACS reagent, ≥99.0%, Sigma-Aldrich), K_2HPO_4 (99+%, for analysis, anhydrous, Thermo Scientific), CH_3CN (LiChrosolv®, isocratic grade for liquid chromatography, Sigma-Aldrich), H_2SO_4 (99.9999%, metal basis, 92% min, Thermo Scientific), $HAuCl_4 \cdot xH_2O$ (99.999% trace metal basis, Sigma-Aldrich), and D_2O (99.97% D, Eurisotop).

S1.2. Copper Electrodes Preparation

Copper-based electrodes (CuE) were prepared by electrodeposition of a thin Cu layer onto pre-cleaned Cu foil in a three-electrode setup, as previously reported.[1]

First, Cu foils were cleaned by sonication for 30 min in ultrapure water and ethanol (EtOH), followed by drying under a N_2 flow. Immediately prior to electrodeposition, the Cu foils were immersed in a 10% HCl solution for 30 s to remove the native surface oxide layer, rinsed thoroughly with ultrapure water, and dried under a N_2 flow.

The pre-cleaned Cu foil was masked with polytetrafluoroethylene (PTFE) tape to define an exposed geometric area of 1 cm^2, which served as the working electrode (WE). A double-junction Ag/AgCl (3 M KCl, Metrohm) and a Pt wire (99.95% metal basis, 1.0 mm diameter, Thermo Scientific) were used as the reference electrode (RE) and counter electrode (CE), respectively, in an undivided electrochemical cell.

An aqueous solution containing 0.05 M $CuSO_4 \cdot 5H_2O$ in 1.5 M H_2SO_4 was used as the electroplating solution. Electrodeposition was carried out galvanostatically by

applying a constant current density of −3 mA cm^{-2} for 40 s while maintaining a fixed distance of 3 cm between the electrodes. After deposition, the CuE electrodes were rinsed with H_2O and dried under a N_2 flow.

S1.3. Electrochemical Measurements

All electrochemical glassware was cleaned in a 10% HNO_3 solution overnight and subsequently rinsed thoroughly with copious amounts of ultrapure water.

A Pt wire (99.95% metal basis, 1.0 mm diameter, Thermo Scientific), previously cleaned in 70% HNO_3 solution and flame-dried, and an Ag/AgCl electrode (3 M KCl, redox.me) were used as CE and RE, respectively. Linear sweep voltammetry (LSV), electrochemical impedance spectroscopy (EIS), and chronoamperometry (CA) experiments were performed using a three-electrode configuration with an Autolab PGSTAT24 potentiostat/galvanostat. All solutions were purged with N_2 for 20 min prior to each measurement.

All reported potentials were converted to the reversible hydrogen electrode (RHE) scale according to Equation 1, where E_{RHE} is the corrected potential versus RHE and $E_{\mathrm{Ag/AgCl}}$ is the applied potential versus Ag/AgCl (3 M KCl). The pH of the electrolyte solutions was measured using a Crison ORP Sension+ PH3 pH meter. For all prepared electrolyte solutions, the pH was 7.0.

$$E_{\mathrm{RHE}} = E_{\mathrm{Ag/AgCl}} + 0.197 + 0.059 \cdot \mathrm{pH} \quad (1)$$

S1.3.1. Impedance Spectroscopy and Active Surface

The electrochemical active surface area (ECSA) was obtained by measuring the frequency dependent impedance in a non-faradaic region of the system from −0.29 to −0.35 V_{RHE}, using impedance spectroscopy between 200 kHz to 20 mHz. The ECSA was calculated from the double layer capacitance (C_{dl}) according to Equation 2, as previously reported by Jaramillo and coworkers,[2] where C_{s} is the specific capacitance of a reference sample.

$$\mathrm{ECSA} = \frac{C_{\mathrm{dl}}}{C_{\mathrm{s}}} \quad (2)$$

S1.3.2. Linear Sweep Voltammetry

The LSV experiments were performed in the absence and presence of 15, 25, 35, and 45 mM acetophenone (ACT) using 10 mL of 0.5 M KCl in a 9:1 H_2O/EtOH mixture as the electrolyte. All LSV measurements were recorded at a scan rate of 1 mV s^{-1} over a potential range from −0.20 to −1.19 V_{RHE}, using CuE as the WE.

S1.3.3. Chronoamperometry Experiments

CA experiments were performed in a three-electrode configuration using a commercial H-type glass cell (Ossila) separated by a previously activated Nafion™ 117 membrane under magnetic stirring. The cathodic compartment contained 15 mL of electrolyte with 25 mM of ACT and the CuE as the WE with an exposed geometrical area of 1 cm^2. The anodic compartment contained a Pt wire as the CE immersed in 15 mL of 0.5 M Na_2SO_3.

All CA experiments were conducted for 4 h unless otherwise stated. Aliquots of 30 μL were withdrawn from the cathodic compartment and diluted in 3 mL of ultrapure water prior to HPLC analysis.

Unless otherwise specified, all electrochemical experiments and product analyses were performed in triplicate to ensure reproducibility.

Conversion, yield, and faradaic efficiency (FE) were calculated using Equations (3)–(5), where z is the number of electrons involved in the electrochemical reaction (z = 2 for ACT → PE), F is the Faraday constant (96485 C), and Q_t is the total charge passed during electrolysis. Carbon balance was obtained according to Equation (6).

$$\%\ \text{Conversion} = \frac{\text{mol of consumed ACT}}{\text{mol of initial ACT}} \cdot 100 \tag{3}$$

$$\%\ \text{Yield} = \frac{\text{mol of PE}}{\text{mol of initial ACT}} \cdot 100 \tag{4}$$

$$\%\ \text{FE} = \frac{\text{mol of PE} \cdot z \cdot F}{Q_t} \tag{5}$$

$$\text{Carbon balance} = \frac{\text{mol of consumed ACT} + \text{mol of PE}}{\text{mol of initial ACT}} \cdot 100 \tag{6}$$

Different conditions were tested using 15 mL of a 25 mM ACT in a 9:1 H_2O/EtOH mixture in the cathodic compartment: (a) applying four different potentials (−0.59, −0.69, −0.79, and −0.89 V_{RHE}) using 0.5 M KCl as electrolyte; (b) varying the electrolyte (0.5 M KCl *vs*. K_2HPO_4/KH_2PO_4) at −0.69 V_{RHE}; (c) varying the halide electrolyte using a 0.5 M K*X* in a 9:1 H_2O/EtOH mixture, where X = Cl^-, Br^-, or I^-, at −0.69 V_{RHE}; (d) varying the ACT concentration (15, 25, 35, and 45 mM) in 0.5 M KCl at −0.69 V_{RHE}.

S1.4. Nafion™ 117 Membrane Activation

The Nafion™ 117 membrane (0.180 mm thick, ≥0.90 meq/g exchange capacity, Sigma-Aldrich) was activated by heating at 80 ºC for 1 h each in the following solutions: 3% H_2O_2, ultrapure water, 2 M H_2SO_4, and ultrapure water. After activation, the membranes were stored in ultrapure water.

S1.5. Characterization Techniques

S1.5.1. HPLC Quantification

Separation and quantification of the products from the CA experiments were performed using a high-performance liquid chromatography (HPLC) Agilent 1260 Infinity II Quaternary System equipped with a 1260 DAD detector. Separation was carried out using an InfinityLab Poreshell 120 EC-C18 column (4.6×100 mm, particle size 2.7 µm, Agilent) maintained at 35 ºC with an injection volume of 10 μL.

Isocratic elution was performed using a mobile phase consisting of 35% acetonitrile (isocratic grade for liquid chromatography LiChrosolv®, Sigma-Aldrich) and 65% 5 mM H_2SO_4 solution (99.9999% metals basis, ≥92%, Thermo Scientific Chemicals) at a flow rate of 0.5 mL min^{-1} and 0.1 mL min^{-2} flux gradient. The elution time for each sample was 24 min.

Compound identification and quantification were performed using commercially available standards. Calibration curves were obtained by correlating the maximum absorbance at selected wavelengths with the concentrations of ACT and PE (Figure S4). The limit of detection (LOD) and limit of quantification (LOQ) for the calibration method were calculated from Equations (7)-(8), where S is the slope obtained from the calibration curve and σ represents the standard deviation of the response.[3,4]

$$\mathrm{LOD} = \frac{3.3 \cdot \sigma}{S} \tag{7}$$

$$\mathrm{LOQ} = \frac{10 \cdot \sigma}{S} \tag{8}$$

S1.5.2. Scanning Electron Microscopy

The surface morphology of the electrodes was examined by scanning electron microscopy coupled with energy-dispersive X-ray spectroscopy (SEM-EDX) on a JEOL 7001F microscope operating at 15 kV and equipped with a Leica Zeiss LEO 440 detector.

S1.5.3. X-Ray Diffraction Pattern

X-ray diffraction patterns (XRD) were recorded using a D8 Advance Brucker-AXS wide-angle diffractometer. Measurements were performed in grazing incidence mode over a 2θ range from 10 to 70º, with a step size of 0.050º and a counting time of 3 s per step.

S1.5.4. Micro Gas Chromatography

Hydrogen detection was performed using a Micro GC 490 (Agilent Technologies) coupled to a closed electrochemical H-cell.

S1.5.5. Electrochemical in situ Surface-Enhanced Raman Spectroscopy (EC-SERS)

SERS experiments were carried out using a WITec alpha300 apyron Raman spectrometer equipped with a 785 nm excitation laser operating at 50 mW. A Zeiss 20× objective was used for in situ measurements conducted in a commercial three-electrode Raman cell (Redox.me) connected to an Autolab PGSTAT204 potentiostat/galvanostat. Typical spectral acquisition times were 20-30 s. Spectra were first collected at the most positive potential and subsequently recorded while progressively stepping to more negative potentials in a chronoamperometric sequence, with each step lasting 2 min. Cosmic ray removal was applied during spectral acquisition.

S1.5.6. Nuclear Magnetic Resonance Spectroscopy (NMR)

^{1}H NMR spectra were collected on a 400 MHz Bruker spectrometer. Samples were obtained from the cathode compartment by taking aliquots of 100 μL and diluted in 400 μL D_2O in an NMR tube prior to analysis.

S1.5.7. Contact Angle Measurements

Contact angles were measured on a Krüss DSA25 Drop Shape Analyzer using a manual drop deposition.

S2. Estimation of the Electrochemical Active Surface Area of CuE Electrodes

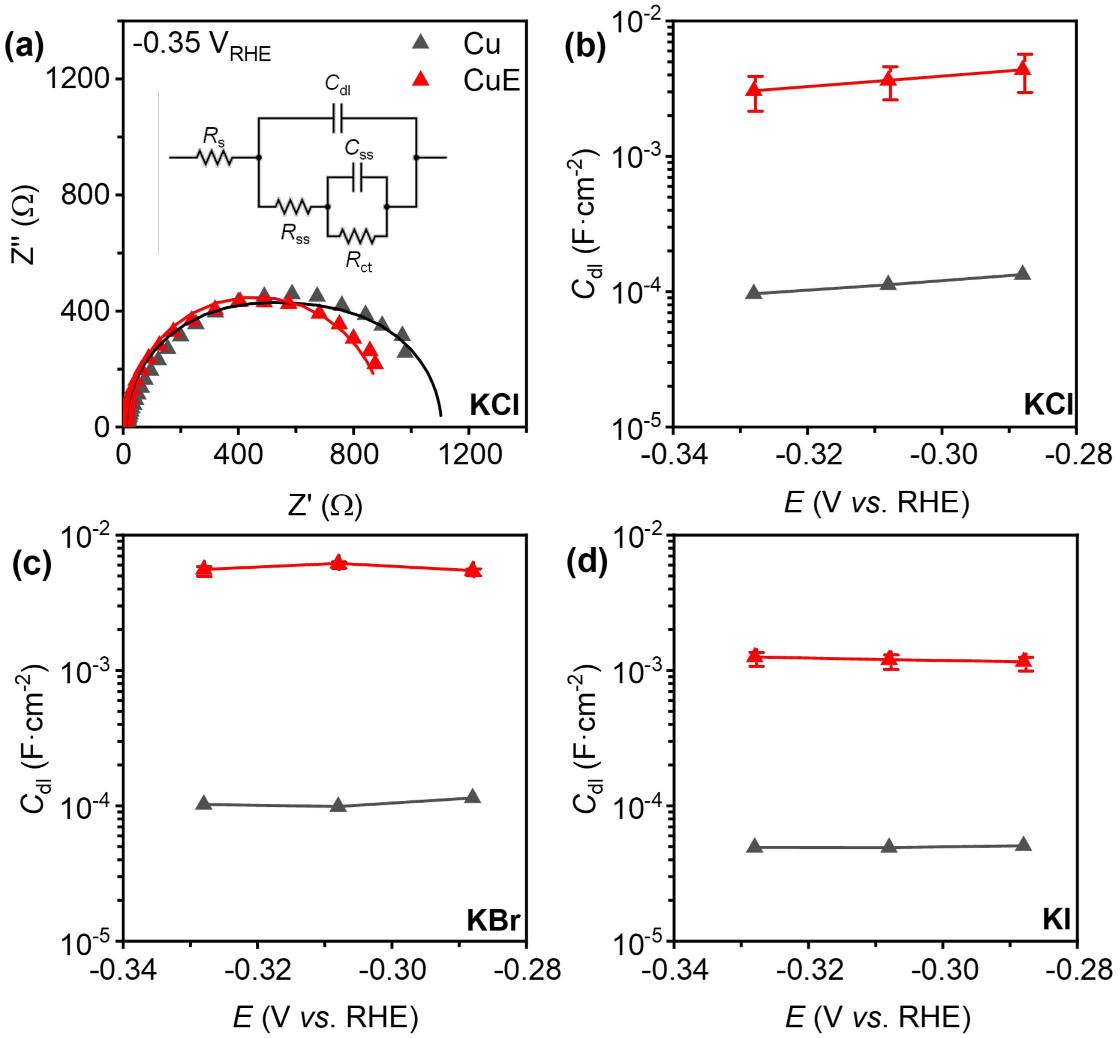

Figure S1. **(a)** Nyquist plots for a polycrystalline Cu foil electrode (Cu) and Cu foil electrode after electrodeposition (CuE) recorded in 0.5 M KCl (9:1 H_2O/EtOH) at -0.35 V_{RHE} in the frequency range from 200 kHz to 20 mHz. This potential lies in a region where no faradaic processes occur. The inset shows the modified Randles circuit used for fitting the impedance data. **(b-d)** Double-layer capacitance (C_{dl}) values obtained for the CuE electrodes in different electrolytes: **(b)** KCl, **(c)** KBr and **(d)** KI, for the estimation of the electrochemical active surface area, compared to Cu foil as reference. The legend in panel **(a)** applies to graphs **(b-d)** as well.

S3. Control Experiments for the Co-solvent Stability

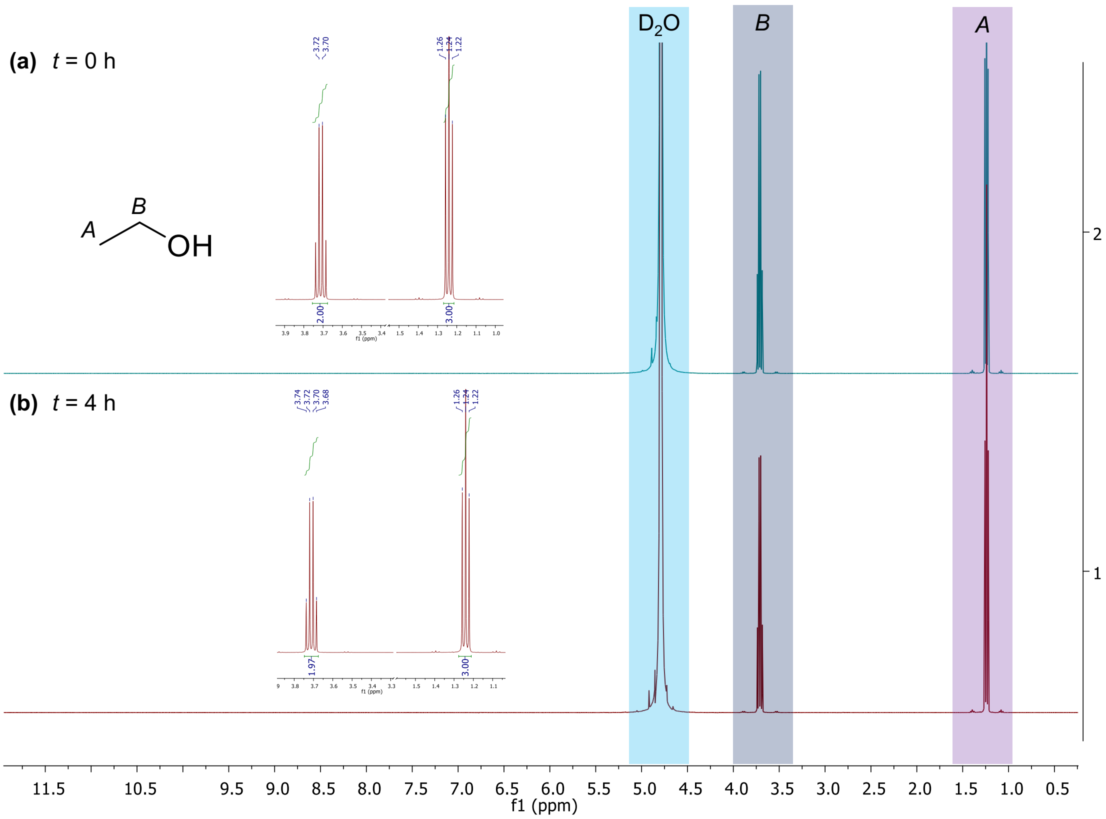

Figure S2. ^{1}H NMR spectra of the blank 9:1 H_2O/EtOH electrolyte solution, recorded **(a)** before and **(b)** after electrolysis under standard electrochemical hydrogenation conditions (–0.69 V_{RHE}, 0.5 M KCl, 4 h) in the absence of acetophenone and CuE electrodes. No detectable changes in the ethanol resonances are observed, indicating that ethanol remains chemically stable under the operating conditions. The inset in both spectra shows a close-up of the ethanol signals. ^{1}H NMR (400 MHz, D_2O) δ (ppm): 1.24 (*t*, -CH_2-), 3.70 (*q*, CH_3-). Notation: *t* triplet, *q* quartet.

S4. Product Separation and Quantification by HPLC

Table S1. Retention times and UV maximum absorption wavelength (λ_{max}) for ACT reduction products and major by-products determined by the HPLC separation method.

**Name**	**Structure**	**Retention time (min)**	**λ_{max} (nm)**
Acetophenone (ACT)	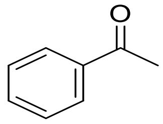	7.19	250
1-Phenylethanol (PE)	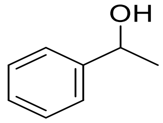	4.05	210
2,3-Diphenyl-2,3-butanediol (DPB)	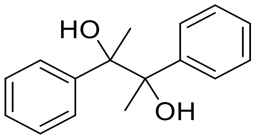	22.25	210
Ethylbenzene (ETB)	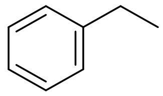	6.72	195

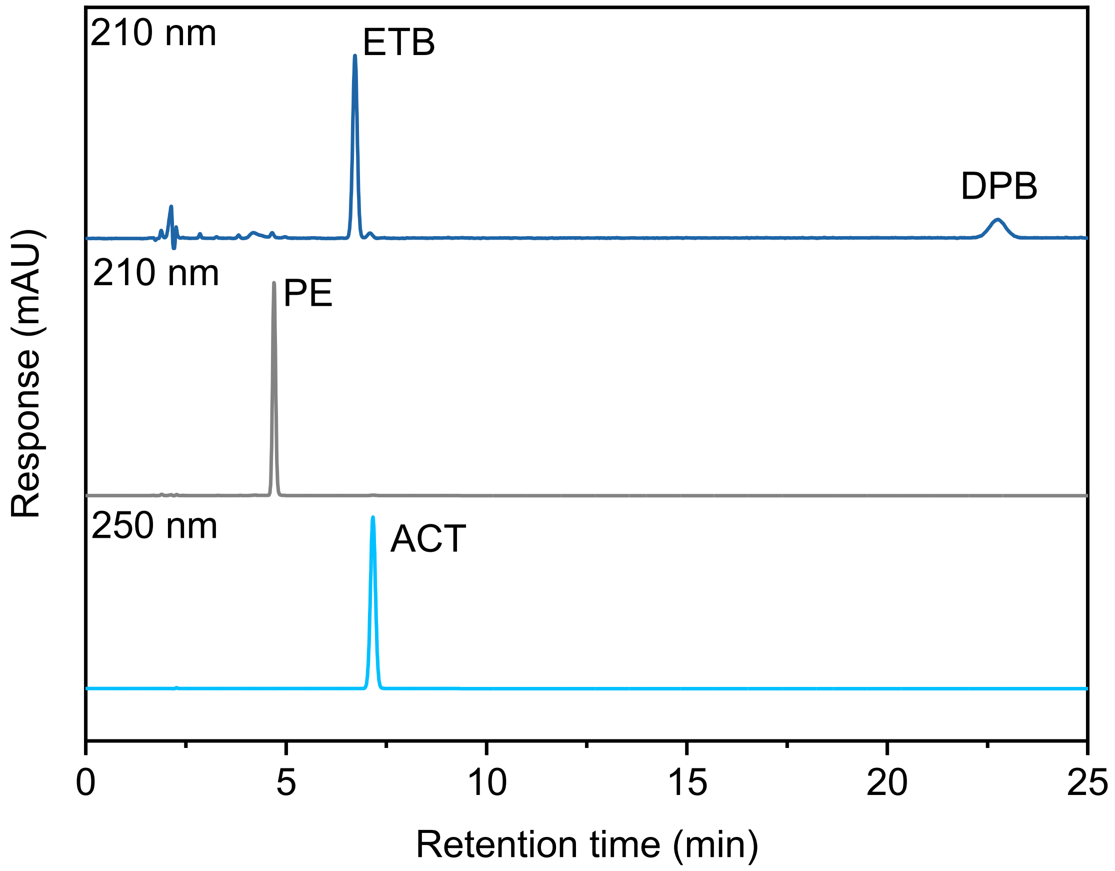

Figure S3. HPLC chromatogram of a commercial standard solution containing ACT, PE, ETB, and DPB in 0.5 M KCl (9:1 H_2O/EtOH).

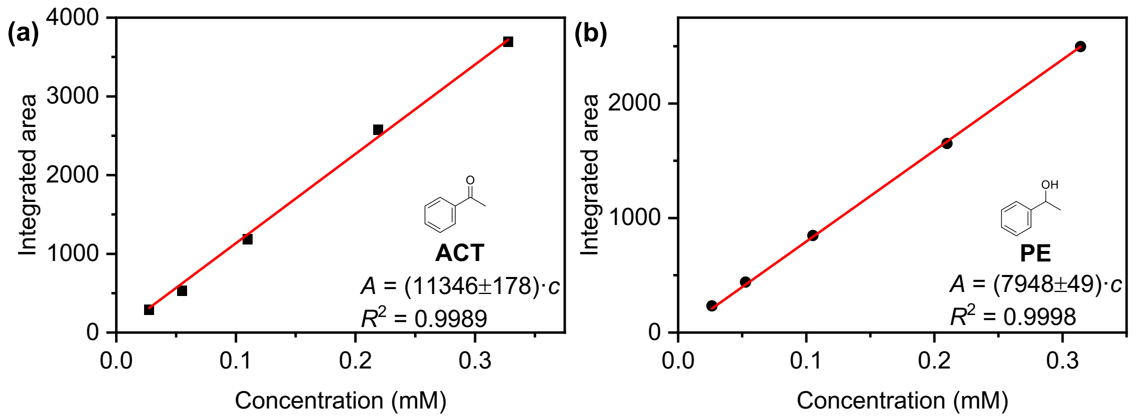

Figure S4. Calibration curves of HPLC used for the quantification of **(a)** ACT and **(b)** PE.

Table S2. Limits of detection (LOD) and quantification (LOQ) obtained for the calibration method.

	**Acetophenone (ACT)**	**1-Phenylethanol (PE)**	**2,3-Diphenyl-2,3-butanediol (DPB)**
LOD (mM)	1.84×10^{-5}	1.78×10^{-4}	1.01×10^{-4}
LOQ (mM)	5.58×10^{-5}	5.41×10^{-4}	3.07×10^{-4}

S5. Acetophenone Electrochemical Hydrogenation Reaction Optimization

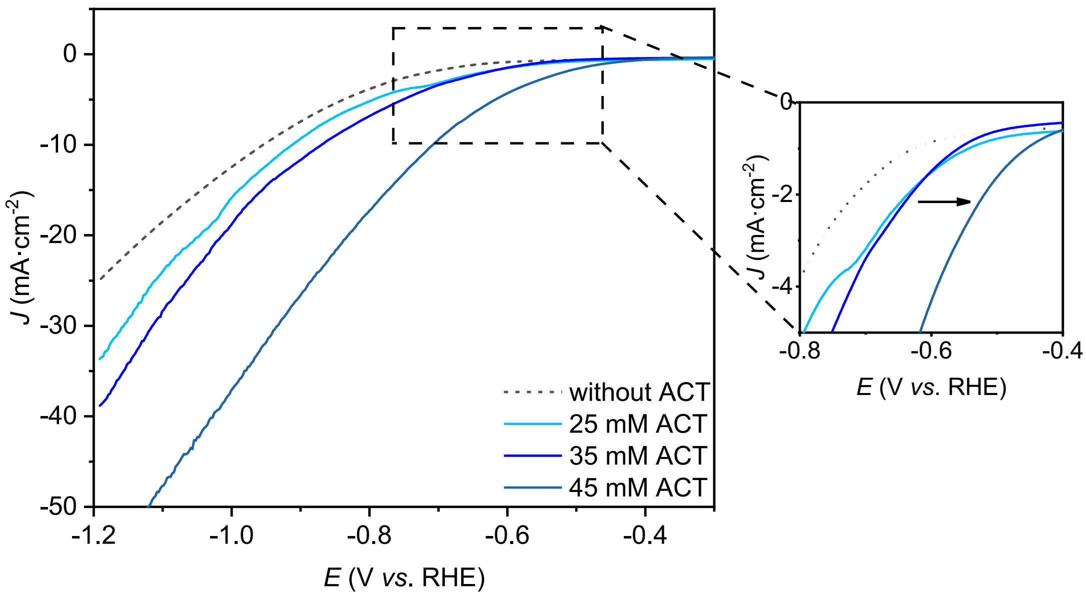

Figure S5. LSV recorded at different ACT concentrations in 0.5 M KCl (9:1 H_2O/EtOH) electrolyte at a scan rate 1 mV s^{-1}. The inset highlights the working potential window and the positive shift in the apparent HER onset with increasing ACT concentration.

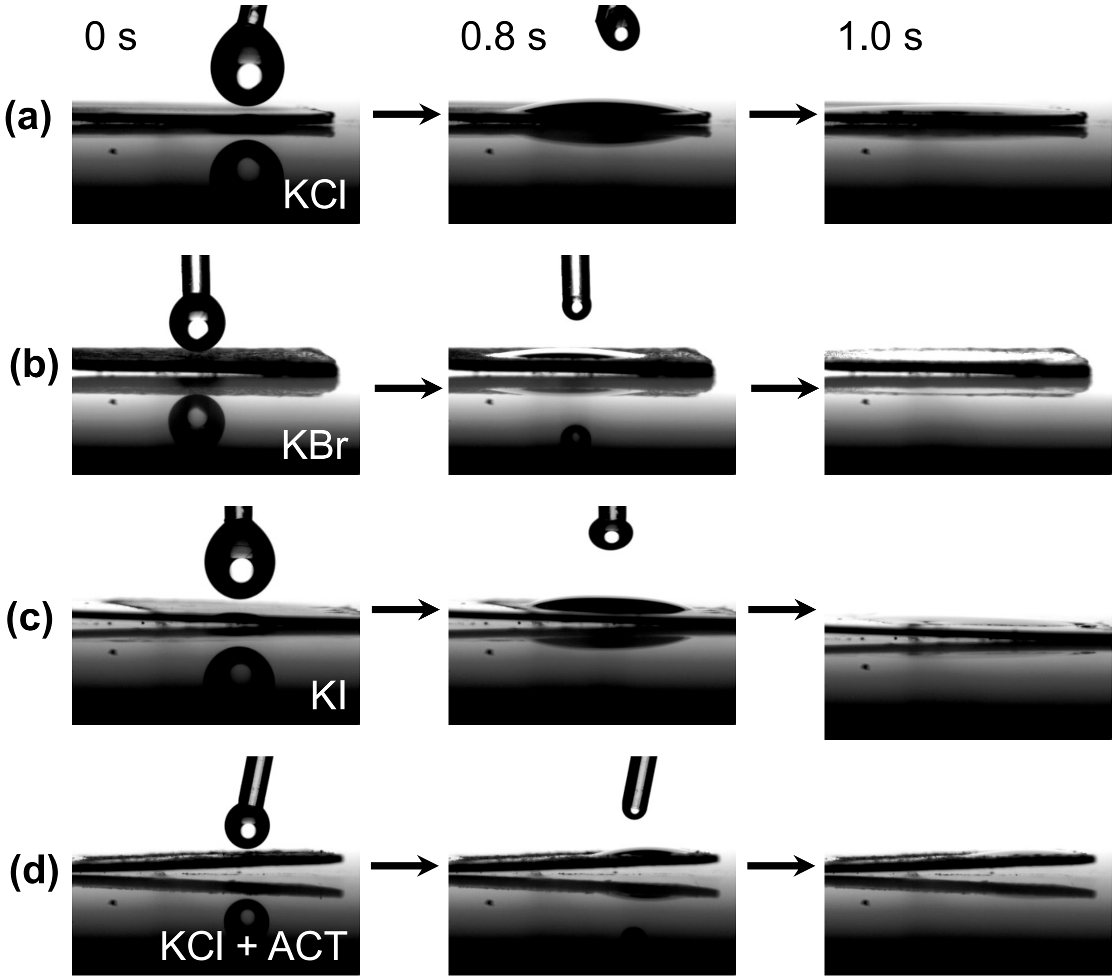

Figure S6. Contact angle for the electrolytes **(a)** KCl, **(b)** KBr, **(c)** KI, and **(d)** 25 mM ACT in KCl on the porous CuE electrode. All electrolytes were prepared at a 0.5 M concentration in 9:1 H_2O/EtOH solution.

Table S3. Carbon balance for the ECH of ACT in 0.5 M electrolyte at −0.69 V_{RHE} over 4 h. All the experiments were done on a constant volume of 15 mL.

**Electrolyte**	***n*(ACT)$_{t=0\ h}$ (mmol)**	***n*(ACT)$_{t=4\ h}$ (mmol)**	***n*(PE)$_{t=4\ h}$ (mmol)**	**Carbon balance (%)**
KCl	0.379±0.003	0.090±0.010	0.270±0.020	96.0±2.0
KBr	0.378±0.004	0.09±0.04	0.26±0.04	92.9±0.7
KI	0.380±0.010	0.27±0.03	0.09±0.03	95±4

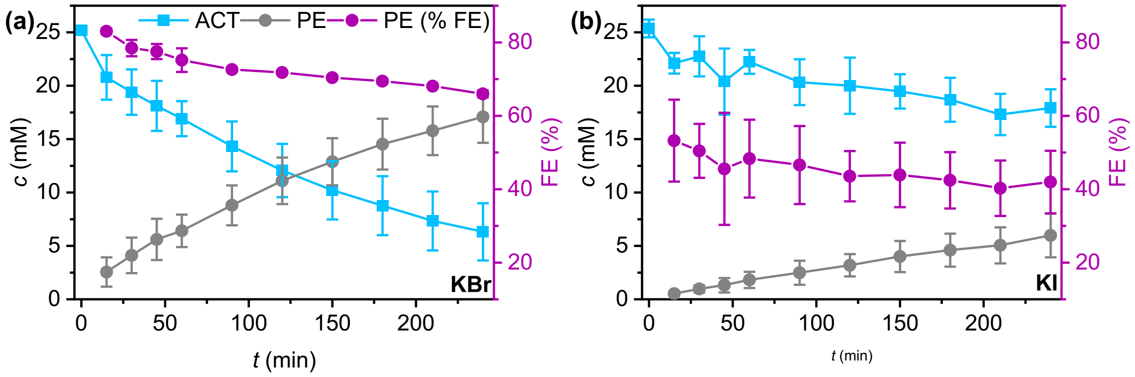

Figure S7. ECH kinetic profiles for 25 mM ACT in 0.5 M **(a)** KBr and **(b)** KI electrolytes at −0.69 V_{RHE} over 4 h. The legend in panel **(a)** also applies to panel **(b)**.

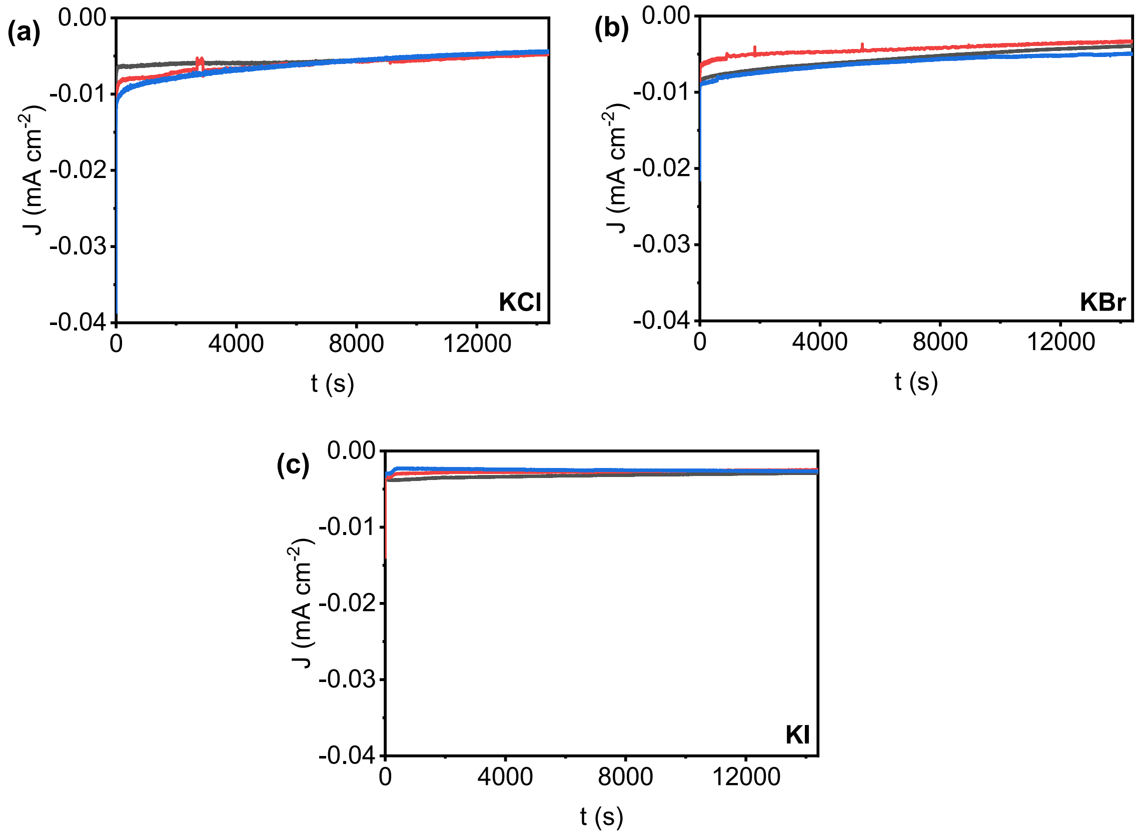

Figure S8. Chronoamperometric curves recorded during electrochemical hydrogenation of 25 mM acetophenone in 0.5 M electrolyte: **(a)** KCl, **(b)** KBr, and **(c)** KI (−0.69 V_{RHE}, 4 h). The stable current overtime supports the reliability of the Faradaic efficiency calculations. The black, red, and blue lines represent the three measured samples for each set of conditions.

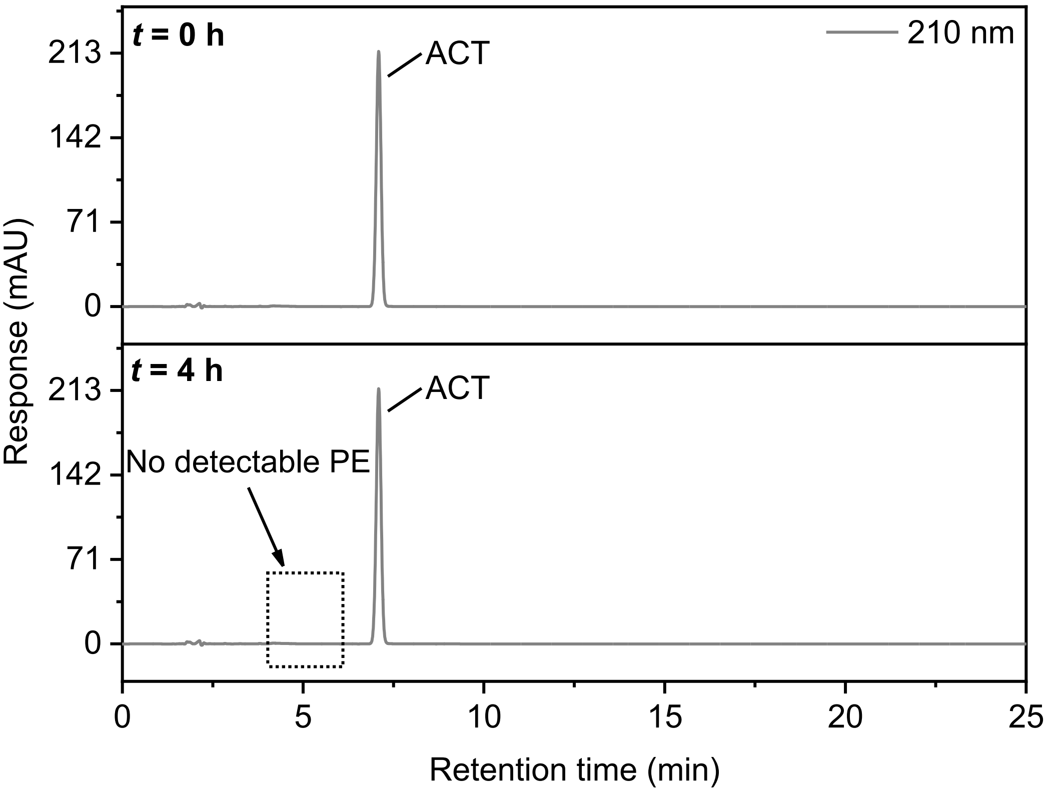

Figure S9. HPLC chromatograms recorded before (t = 0 h) and after (t = 4 h) electrolysis using a planar Cu foil electrode under standard reaction conditions (25 mM ACT in 0.5 M KCl 9:1 H_2O/EtOH at –0.69 V_{RHE}). No formation of hydrogenation products is observed, indicating negligible catalytic activity compared to CuE.

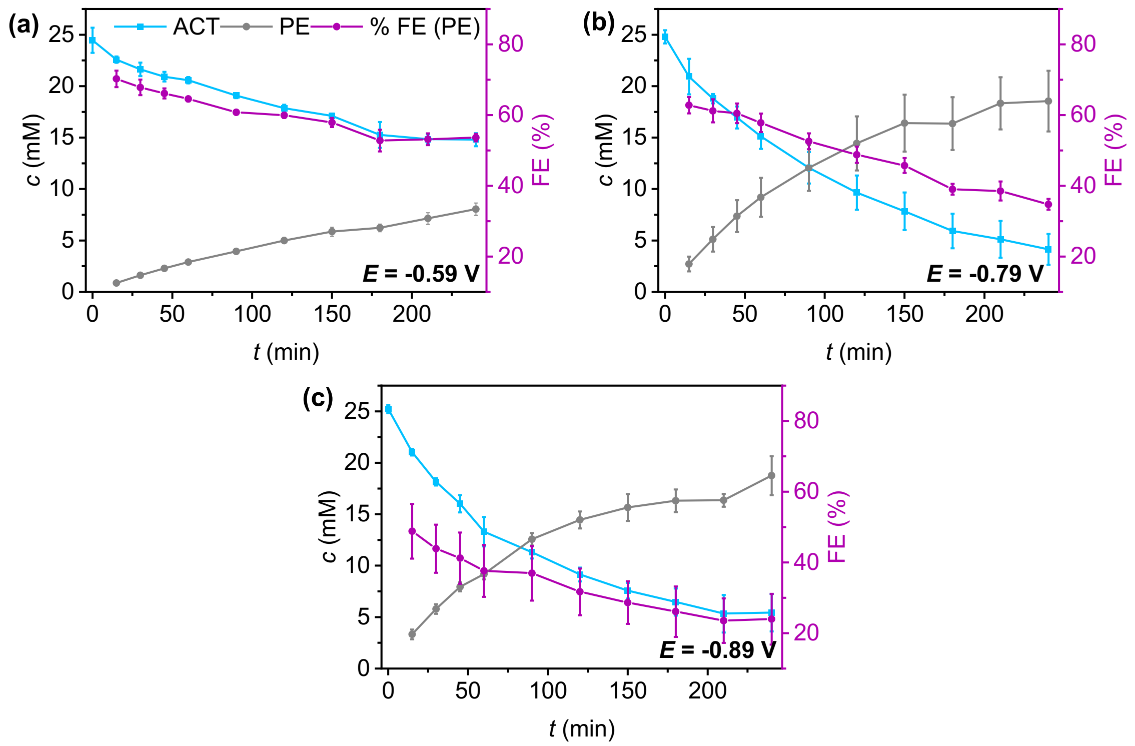

Figure S10. ECH kinetic profiles for 25 mM ACT in 0.5 M KCl (9:1 H_2O/EtOH) at **(a)** −0.59 V_{RHE}, **(b)** −0.79 V_{RHE}, and **(c)** −0.89 V_{RHE}. All reactions were conducted for 4 h. The legend in panel **(a)** applies to panels **(b)** and **(c)**.

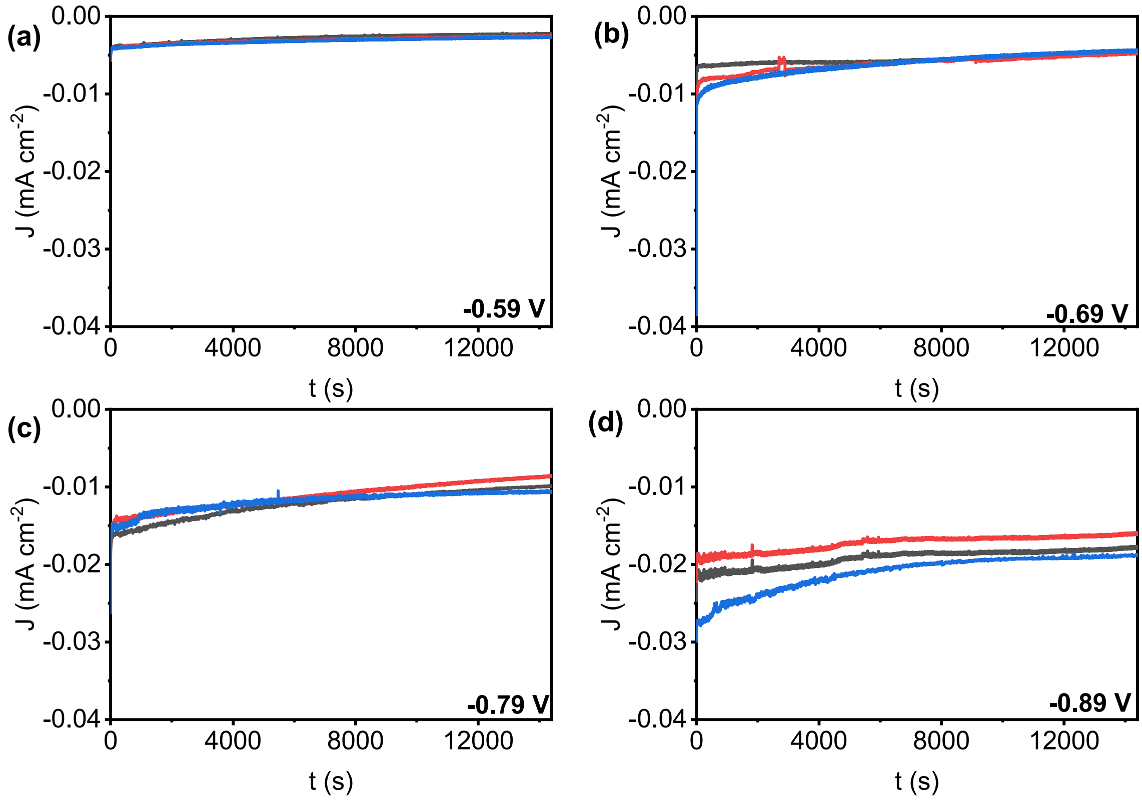

Figure S11. Chronoamperometric curves recorded during electrochemical hydrogenation of 25 mM acetophenone in 0.5 M KCl at different potentials: **(a)** –0.59 V_{RHE}, **(b)** –0.69 V_{RHE}, **(c)** –0.79 V_{RHE} and **(d)** –0.89 V_{RHE}. All reactions were conducted for 4 h. The black, red and blue lines represent the three measured samples for each condition.

Table S4. Carbon balance for the ECH of ACT in 0.5 M KCl at different applied potentials over 4 h. All the experiments were done on a constant volume of 15 mL.

E **(V_{RHE})**	***n*(ACT)$_{t=0\,h}$ (mmol)**	***n*(ACT)$_{t=4\,h}$ (mmol)**	***n*(PE)$_{t=4\,h}$ (mmol)**	**Carbon balance (%)**
–0.59	0.370±0.020	0.222±0.009	0.121±0.009	94±4
–0.69	0.379±0.003	0.090±0.010	0.270±0.020	96.0±2.0
–0.79	0.370±0.010	0.060±0.020	0.29±0.04	92.0±1.0
–0.89	0.379±0.006	0.080±0.020	0.28±0.03	96±3
–0.69	0.640±0.020	0.16±0.03	0.46±0.05	97±4

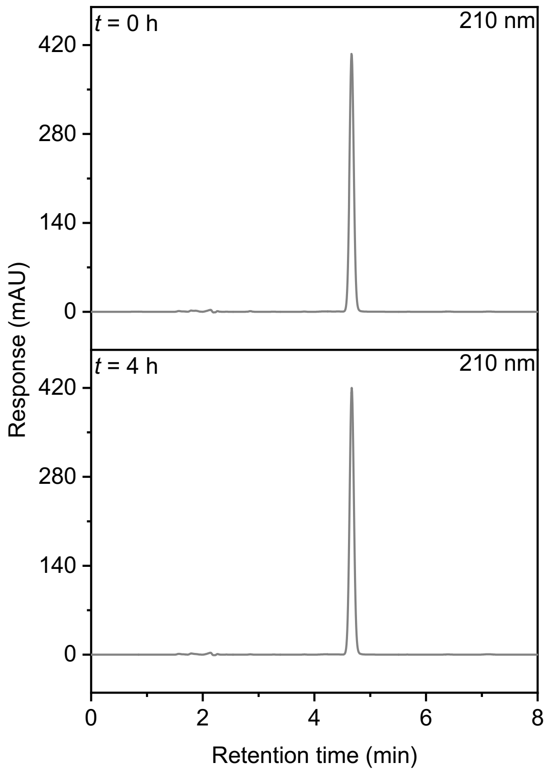

Figure S12. HPLC chromatograms recorded for the ECH of 25 mM PE in 0.5 M KCl (9:1 H_2O/EtOH) at −0.69 V_{RHE} for 4 h using CuE as the working electrode, showing no detectable conversion of PE to ETB.

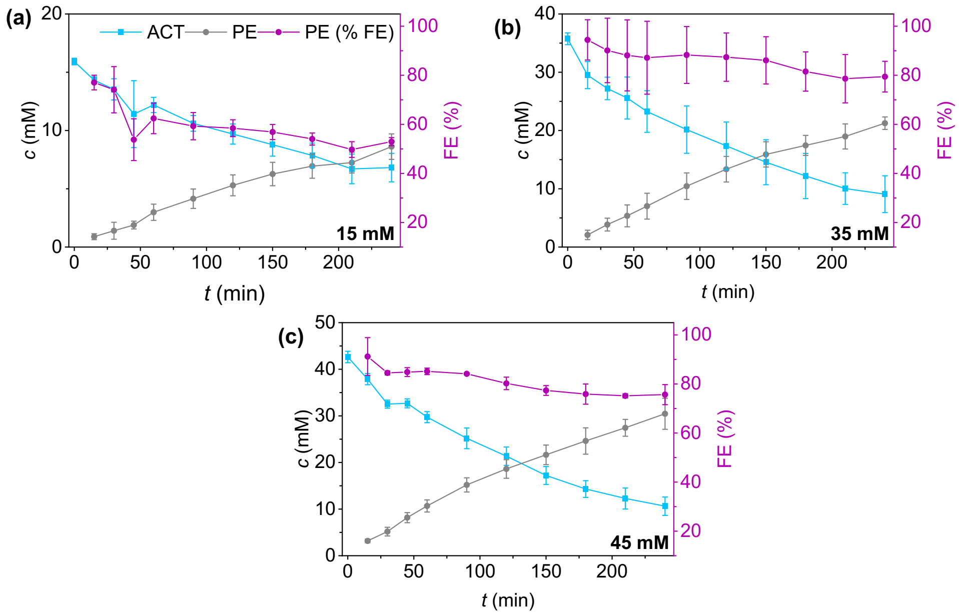

Figure S13. Kinetic profiles for ACT electrochemical hydrogenation at different initial ACT concentrations: **(a)** 15 mM, **(b)** 35 mM, and **(c)** 45 mM in 0.5 M KCl (9:1 H_2O/EtOH) at −0.69 V_{RHE} over 4 h.

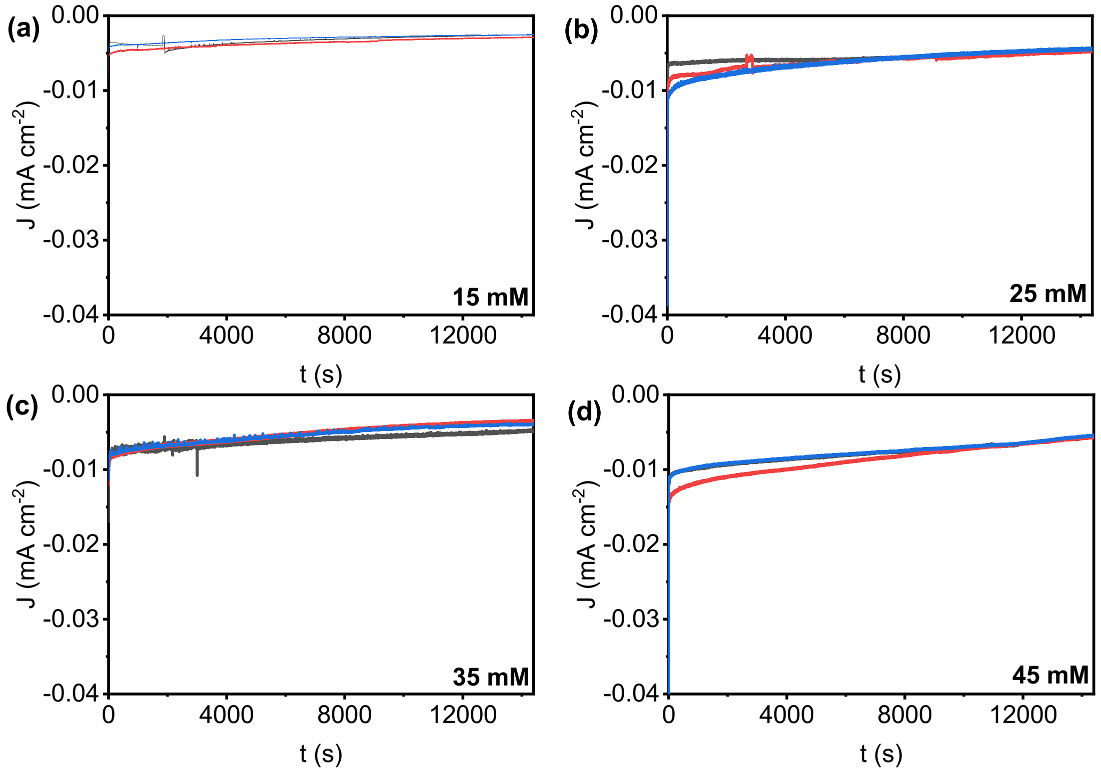

Figure S14. Chronoamperometric curves recorded during electrochemical hydrogenation of acetophenone in 0.5 M KCl at –0.69 V_{RHE} and different concentrations: **(a)** 15, **(b)** 25, **(c)** 35, and **(d)** 45 mM. All reactions were conducted for 4 h. The black, red and blue lines represent the three measured samples for each condition.

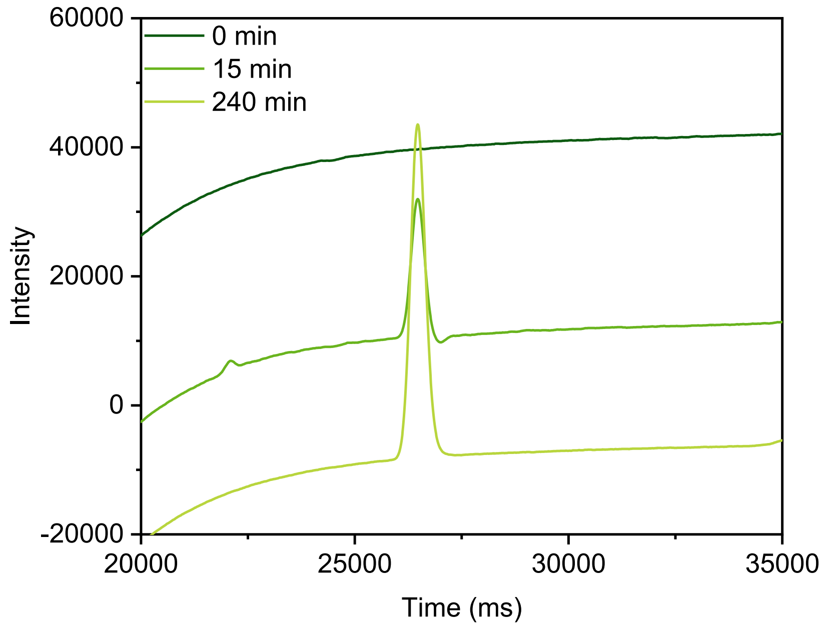

Figure S15. Micro-GC chromatogram for H_2 detection during electrochemical hydrogenation of 45 mM ACT in 0.5 M KCl (9:1 H_2O/EtOH) at −0.69 V_{RHE} for 4 h using CuE as the cathode.

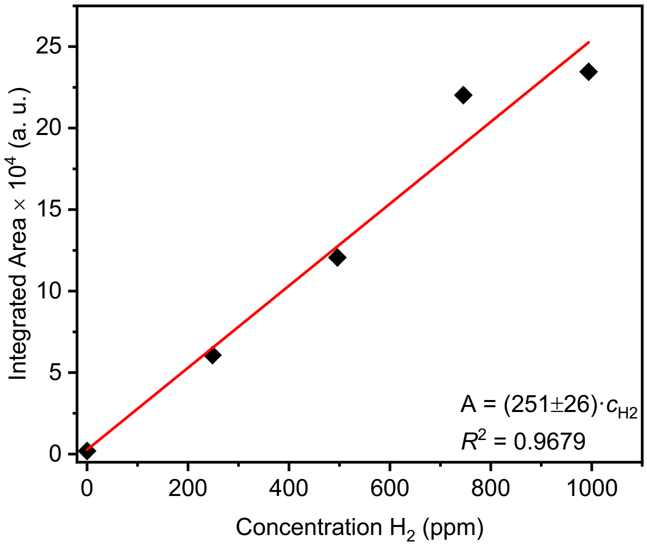

Figure S16. Calibration curve for the quantification of H_2.

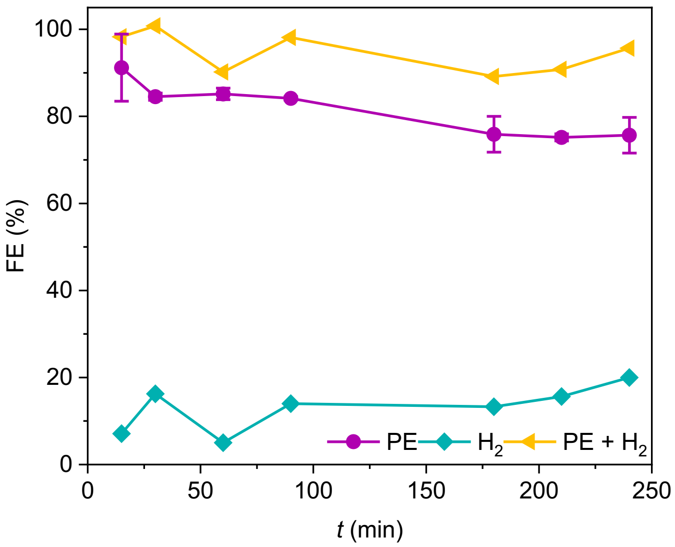

Figure S17. Time-dependent FE toward PE, H_2, and their combined total during electrolysis under optimized conditions (45 mM ACT in 0.5 M KCl at –0.69 V_{RHE}). Minor fluctuations in cumulative FE arise from experimental uncertainty associated with repeated headspace sampling and GC quantification.

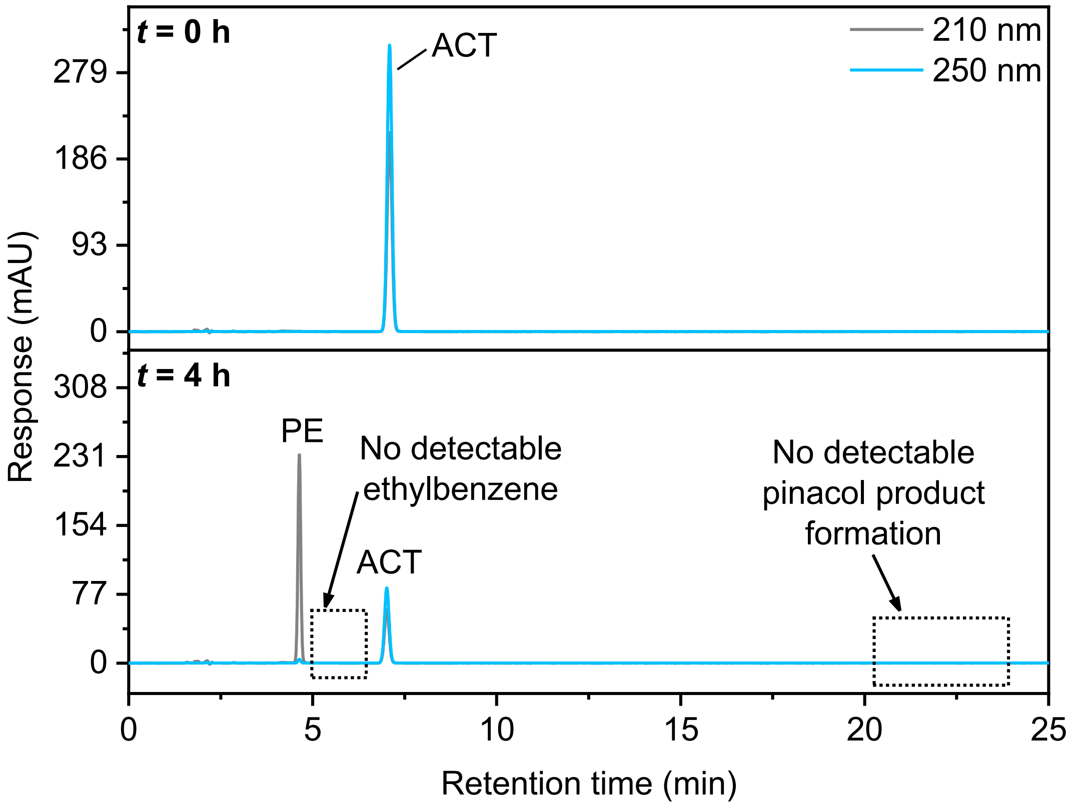

Figure S18. HPLC chromatograms at t = 0 h and t = 4 h during electrochemical hydrogenation of 25 mM ACT in 0.5 M KCl (9:1 H_2O/EtOH) at −0.69 V_{RHE}. Signals recorded at 210 nm (gray) and 250 nm (blue) show the conversion of ACT to PE. No additional peaks corresponding to pinacol coupling or hydrogenolysis products are observed within the detection limits of the method.

S6. Kinetic Analysis for the ECH of ACT

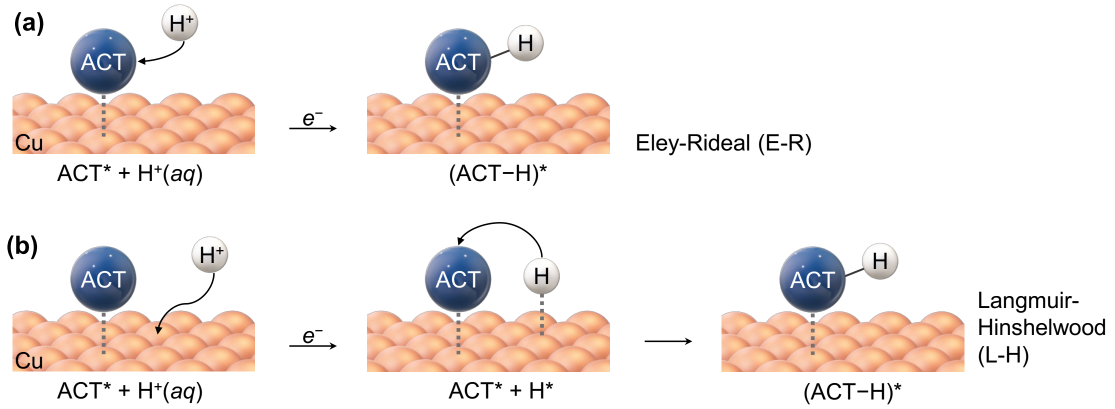

Figure S19. Schematic representations of two surface-catalyzed hydrogenation mechanisms: **(a)** Eley–Rideal (E–R) mechanism and **(b)** Langmuir–Hinshelwood (L–H) mechanisms. Surface-adsorbed species are indicated by *. ACT–H represent a reduced adsorbed intermediate formed during the ECH process.

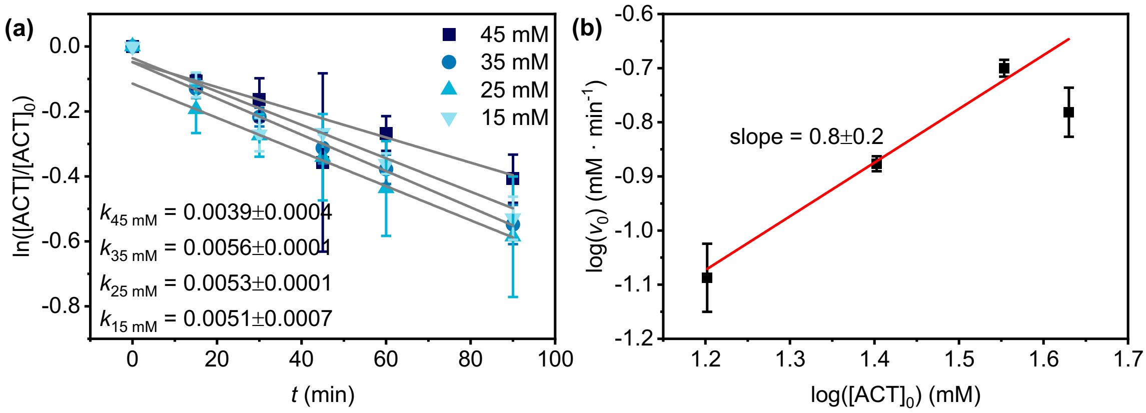

Figure S20. (a) Pseudo-first order kinetic profile for the ECH of ACT showing the calculated rate constant (k) at different ACT concentrations. Errors bars are overlaid. **(b)** Initial conversion rate for the ECH of ACT on CuE as a function of ACT concentration in 0.5 M KCl (9:1 H_2O/EtOH). See fitting equations in Table S5.

Table S5. Fitting equations and R^2 values corresponding to the kinetic plots shown in Figure S20. Pseudo-first-order fits were obtained from plots of $\ln([ACT]/[ACT]_0)$ versus time, and the apparent reaction order was determined from the $\log(r_0)$ versus $\log([ACT]_0)$ relationship.

Figure	$[ACT]_0$ (mM)	Fit equation	R^2
S20a	45	$\ln([ACT]/[ACT]_0) = (-0.0039 \pm 0.0004) \cdot t - (0.048 \pm 0.008)$	0.9844
	35	$\ln([ACT]/[ACT]_0) = (-0.00560 \pm 0.00010) \cdot t - (0.048 \pm 0.005)$	0.9980
	25	$\ln([ACT]/[ACT]_0) = (-0.00530 \pm 0.00010) \cdot t - (0.114 \pm 0.006)$	0.9959
	15	$\ln([ACT]/[ACT]_0) = (-0.0051 \pm 0.0007) \cdot t - (0.04 \pm 0.03)$	0.9237
S20b	-	$\log(r_0) = (0.80 \pm 0.20) - (2.3 \pm 0.4) \cdot \log([ACT]_0)$	0.8217

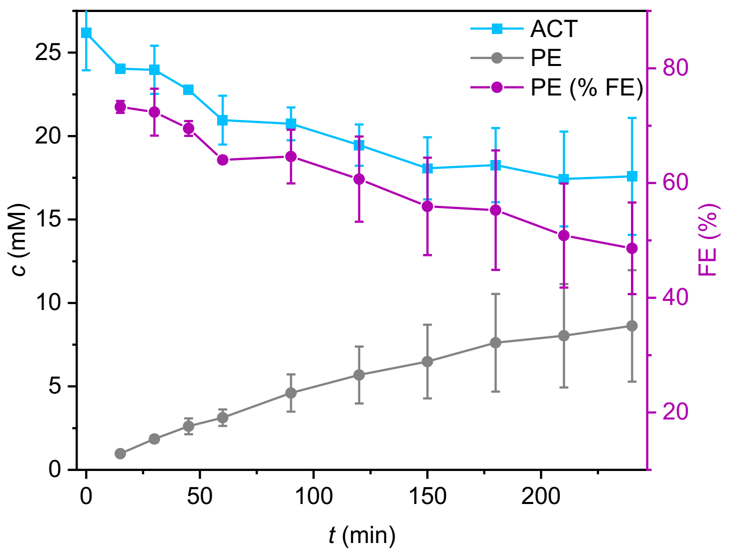

Figure S21. Time-dependent concentration profiles obtained during the quenching experiment performed with 25 mM 2,4-DCP and 25 mM ACT in 0.5 M KCl (9:1 H_2O/EtOH) at −0.69 V_{RHE} over 4 h.

S7. Electrochemical in situ Surface-Enhanced Raman Spectroscopy Results

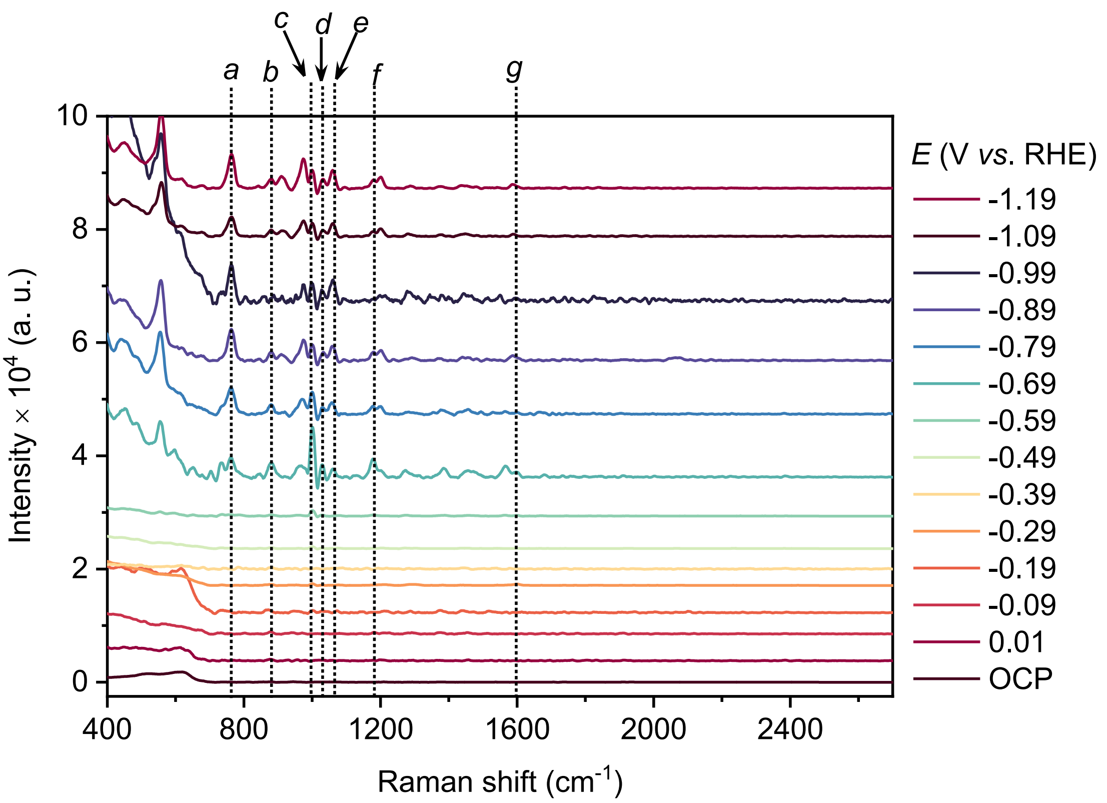

Figure S22. Raman spectra recorded at different applied potentials, starting at open-circuit potential (OCP) and progressing to −1.19 V_{RHE}. To enhance the Raman signal, a Cu foil was first immersed in a 10 mM $HAuCl_4 \cdot xH_2O$ solution for 10 s to deposit a small amount of Au via galvanic replacement. A thicker Cu layer was subsequently electrodeposited following the procedure described in Section S1.2. Signal assignments: **(a)** $\rho(CH_3)$, **(b)** γ(C–H), **(c)** Breathing mode, **(d)** ν(C–O), **(e)** ν(C–C), **(f)** δ(C–H) and **(g)** ν(C=O) (ν: stretching; ρ: rocking; γ: out-of-plane bending; δ: in-plane bending).

S8. Computational Methods

Periodic density functional theory (DFT) calculations were performed using the Vienna Ab Initio Simulation Package (VASP, version 6.4.2).[5–8] The Bayesian error estimation functional with van der Waals dispersions (BEEF-vdW) was used as the exchange-correlation functional.[9–11] Methodological specifications were based on previous work on ACT electroreduction[1] and related theoretical studies.[12,13]

Plane waves with a kinetic energy cut-off of 500 eV were used to represent valence electrons, while core electrons were described using projector augmented-wave (PAW) pseudopotentials.[14] Convergence thresholds for electronic and ionic steps were set to 10^{-6} eV and 0.01 Å eV^{-1}, respectively. Structural relaxations were performed using the conjugate-gradient method with a step size of 0.1 Å.

Gas-phase molecules were optimized at the Γ-point using cubic simulation cells with 15 Å of vacuum to prevent spurious interactions. For slab calculations, Monkhorst-Pack k-point grids corresponding to a density of 0.17 $points^{-1} \cdot Å^{-1}$ in the x and y directions and a single k-point along the non-periodic z direction were used for surface–adsorbate structures. In the direction normal to the surface, sufficient vacuum spacing was introduced to eliminate spurious interactions. Electronic occupancies for gas-phase molecules were treated using Gaussian smearing (0.05 eV width), whereas first-order Methfessel–Paxton smearing (0.2 eV width) was used for surface calculations.[15]

All structures were generated using the Atomic Simulation Environment (ASE) software.[16] The Cu(111) facet used in this work was modeled following previous studies.[1] In brief, $p(2\times2)$ and $p(4\times4)$ surface supercells were constructed from bulk *fcc* Cu (mp-30, lowest energy above hull, lattice parameter 3.62 Å) obtained from the Materials Project database[17] and subsequently optimized using a Birch-Murnaghan equation of state fitting.[18] The optimized lattice parameter for Cu was 3.66 Å. The Cu(111) slab consisted of four atomic layers, with the bottom two layers fixed at their bulk positions during structural optimization.

Gas-phase vibrational frequency calculations were performed by displacing all atoms, whereas only adsorbates atoms (*H, *Cl, *Br, and *I) were displaced for surface slabs. The finite-difference method was employed using an atomic displacement of ±0.02 Å. Gibbs energy corrections were calculated at 298.15 K and 1 atm using the VASPkit implementation of the ideal gas and harmonic oscillator approximations for gas-phase molecules and adsorbed species, respectively.[19]

Vibrational frequencies below 50 cm^{-1} were set to this value, while small spurious imaginary frequencies were replaced by real frequencies of 12 cm^{-1} following the approach proposed by Nørskov and co-workers.[20,21]

The gas-phase Gibbs binding energies of n *H and m halide adsorbates (X = *Cl, *Br, *I) were calculated at 0 V_{RHE} ($\Delta G_{(n\,{*}\mathrm{H},m\,{*}\mathrm{X})}(0_{\mathrm{RHE}})$) using the computational hydrogen electrode (CHE) model, with H_2 and X_2 molecules as references. This approach follows the methodology described by Groß and co-workers, Equation (9).[22,23]

$$\Delta G_{(n\,{*}\mathrm{H},m\,{*}\mathrm{X})}(0_{\mathrm{RHE}}) = G_{(n\,{*}H,m\,{*}X)} - \left(G_{*} + \frac{n}{2}\,G_{\mathrm{H}_2} + \frac{m}{2}\,G_{\mathrm{X}_2}\right) + m\,e\,E°(\mathrm{X}_2/\mathrm{X}^-) \qquad (9)$$

Where $G_{(n\,{*}\mathrm{H},m\,{*}\mathrm{X})}$ is the Gibbs energy of the adsorbed species containing n hydrogen atoms and m halide atoms, G_{*} is the Gibbs energy of the bare metal surface, and G_{X_2} and G_{H_2} are the Gibbs energies of halogen and hydrogen molecules, respectively. $E°(\mathrm{X}_2/\mathrm{X}^-)$ is the standard reduction potential of the corresponding halide redox couple (**Table S6**). Halide activities were set to $a_{\mathrm{X}^-} = 1$ following the methodological approach described above.

Table S6. Standard reduction potentials of the halide redox couples following the methodology of Groß and co-workers.[23]

Redox couple	$E°(\mathrm{X}_2/\mathrm{X}^-)$ (V *vs* SHE)
$\frac{1}{2}\mathrm{Cl}_2 + e^- \rightleftarrows \mathrm{Cl}^-$	+1.36
$\frac{1}{2}\mathrm{Br}_2 + e^- \rightleftarrows \mathrm{Br}^-$	+1.09
$\frac{1}{2}\mathrm{I}_2 + e^- \rightleftarrows \mathrm{I}^-$	+0.54

Surface coverage analyses based on adsorption Gibbs energies at non-zero potentials versus RHE, $\Delta G_{(n\mathrm{H},m\mathrm{X})}(E)$, were obtained using Equation (10):

$$\Delta G_{(n\,{*}\mathrm{H},m\,{*}\mathrm{X})}(E) = \Delta G_{(n\,{*}\mathrm{H},m\,{*}\mathrm{X})}(0_{\mathrm{RHE}}) + (n - m)eE \qquad (10)$$

Because different slab sizes were used to probe a wider range of surface coverages, adsorption Gibbs energies were normalized per surface Cu atom (N) according to Equation (11):

$$\Delta g_{(n\,^*\mathrm{H},m\,^*\mathrm{X})}(E) = \frac{\Delta G_{(n\,^*\mathrm{H},m\,^*\mathrm{X})}(E)}{N} \tag{11}$$

For $p(2\times2)$ and $p(4\times4)$ slabs, $N = 4$ and $N = 16$, respectively.

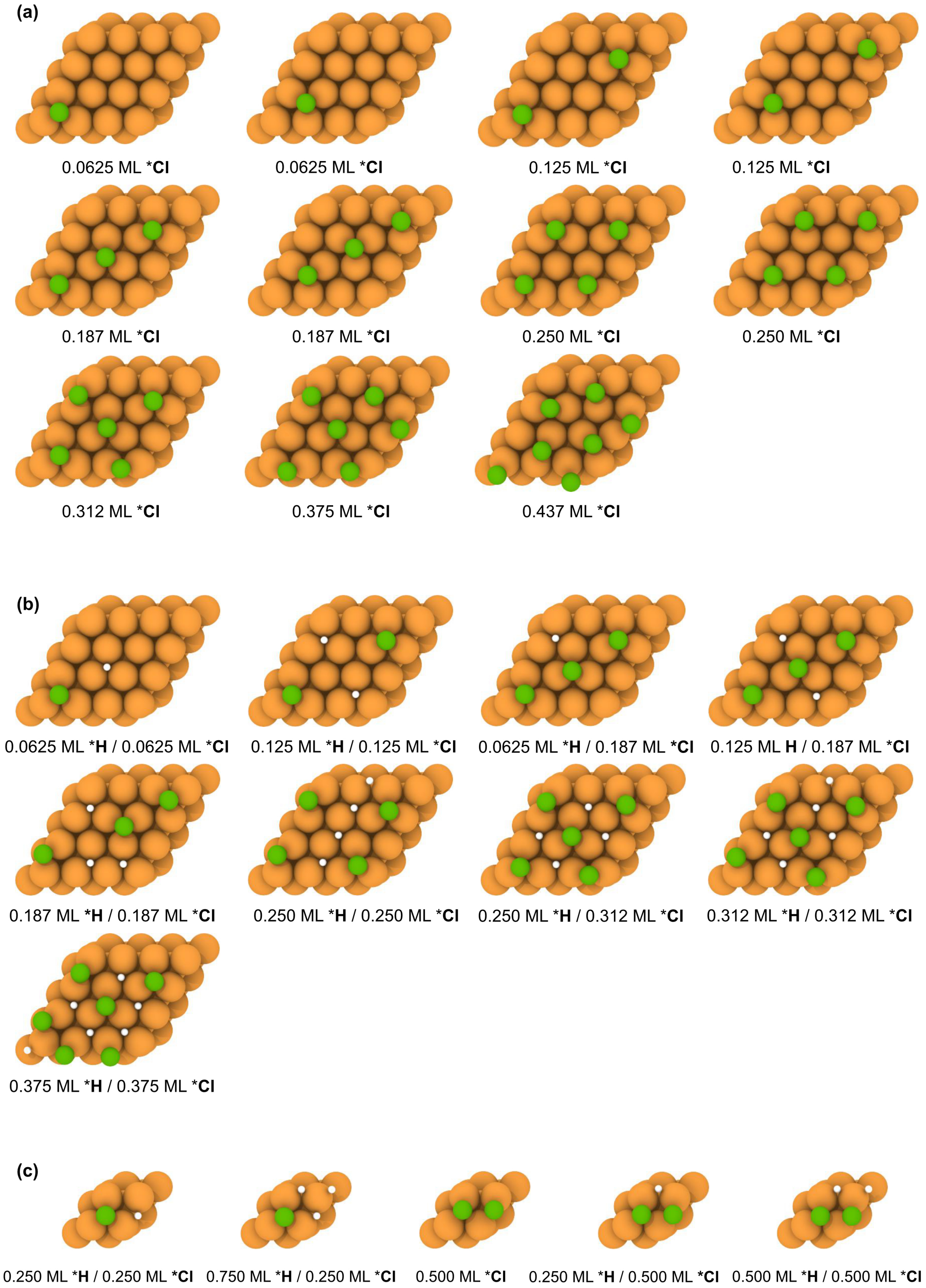

Figure S23. Optimized geometries of the *p*(4×4) Cu(111) slab with different surface coverages of **(a)** chloride, **(b)** co-adsorbed chloride–hydrogen species, and (c) both adsorbates on the smaller p(2×2) Cu(111) slab. Monolayer fractional coverages are indicated below each structure.

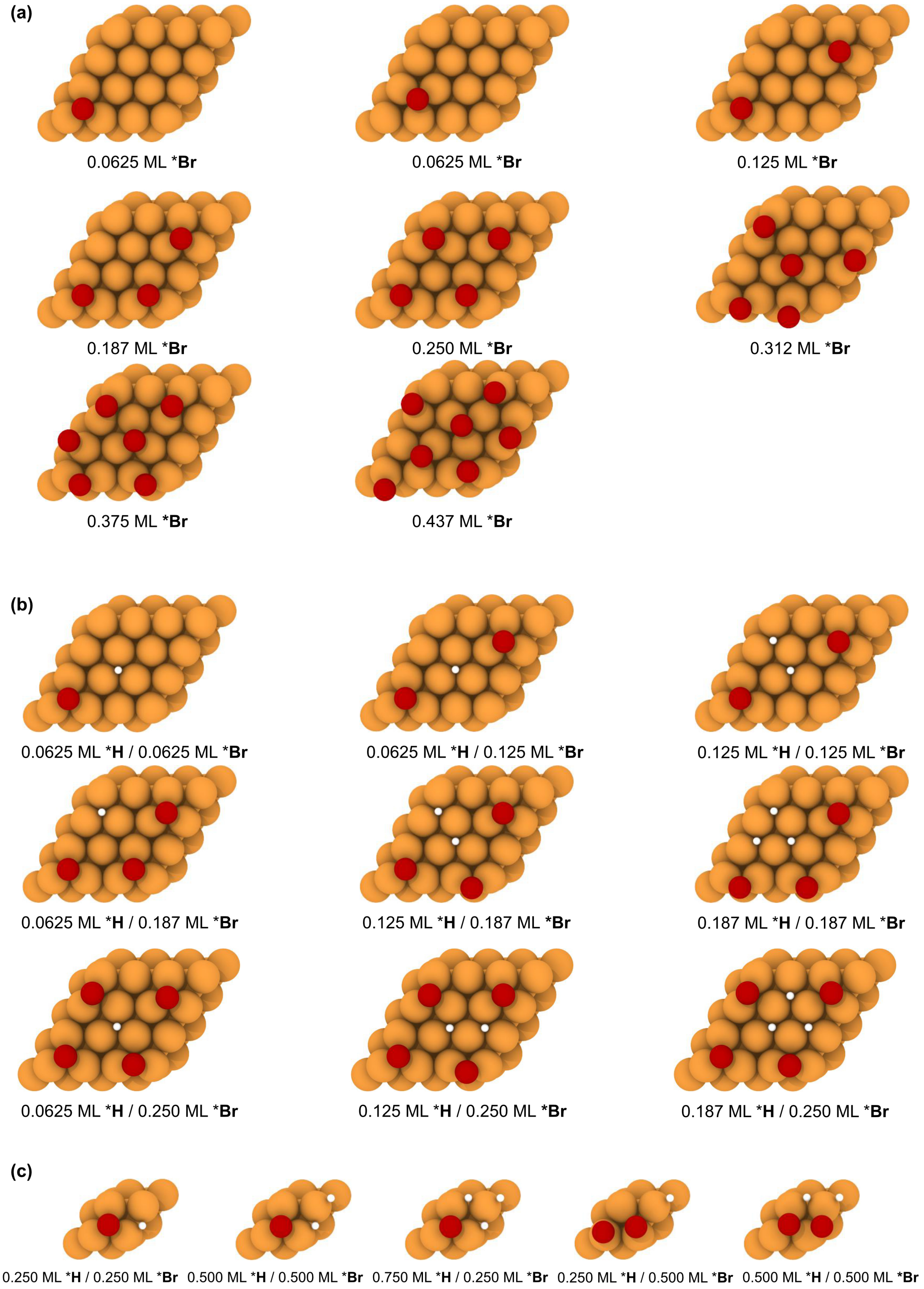

Figure S24. Optimized geometries of the *p*(4×4) Cu(111) slab with different surface coverages of **(a)** bromide, **(b)** co-adsorbed bromide–hydrogen species, and **(c)** both adsorbates on the smaller *p*(2×2) Cu(111) slab. Monolayer fractional coverages are indicated below each structure.

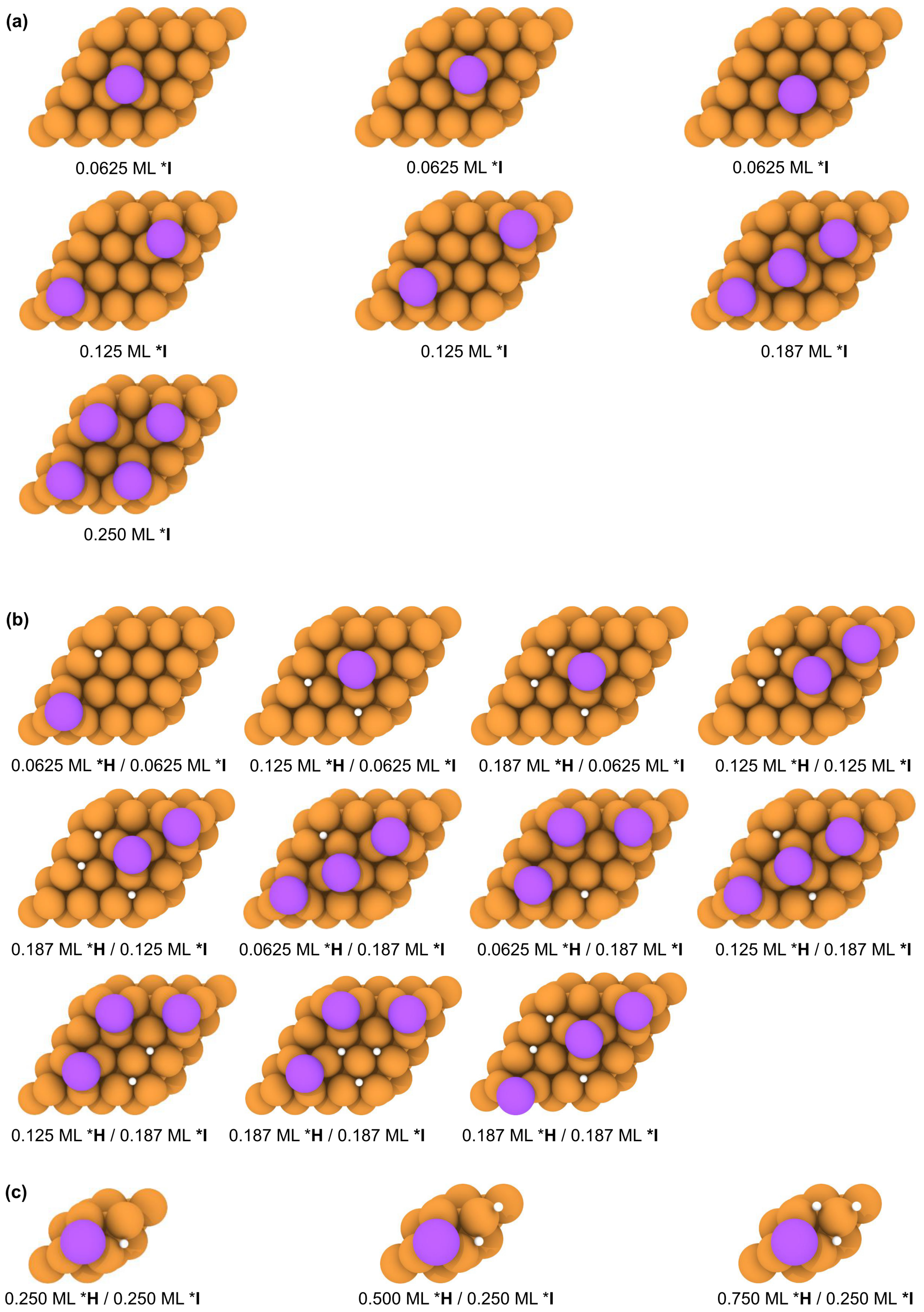

Figure S25. Optimized geometries of the $p(4\times4)$ Cu(111) slab with different surface coverages of **(a)** iodide, **(b)** co-adsorbed iodide–hydrogen, and **(c)** both adsorbates on the smaller $p(2\times2)$ Cu(111) slab. Monolayer fractional coverages are indicated below each structure.

S9. References

(1) Ciotti, A.; Rahaman, M.; Yeung, C. W. S.; Li, T.; Reisner, E.; García-Melchor, M. Driving Electrochemical Organic Hydrogenations on Metal Catalysts by Tailoring Hydrogen Surface Coverages. *J. Am. Chem. Soc.* **2025**, *147*, 13158–13168. https://doi.org/10.1021/jacs.4c15821.

(2) McCrory, C. C. L.; Jung, S.; Peters, J. C.; Jaramillo, T. F. Benchmarking Heterogeneous Electrocatalysts for the Oxygen Evolution Reaction. *J. Am. Chem. Soc.* **2013**, *135* (45), 16977–16987. https://doi.org/10.1021/ja407115p.

(3) Vial, J.; Jardy, A. Experimental Comparison of the Different Approaches to Estimate LOD and LOQ of an HPLC Method. *Anal. Chem.* **1999**, *71* (14), 2672–2677. https://doi.org/10.1021/ac981179n.

(4) Miller, J. N.; Miller, J. C. *Statistics and Chemometrics for Analytical Chemistry*, 6th ed.; Pearson Education: Harlow, 2010.

(5) Hohenberg, P.; Kohn, W. Inhomogeneous Electron Gas. *Physical Review* **1964**, *136* (3B), B864–B871.

(6) Kohn, W.; Sham, L. J. Self-Consistent Equations Including Exchange and Correlation Effects. *Physical Review* **1965**, *140* (4A), A1133–A1138.

(7) Kresse, G.; Furthmüller, J. Efficiency of Ab-Initio Total Energy Calculations for Metals and Semiconductors Using a Plane-Wave Basis Set. *Comput. Mater. Sci.* **1996**, *6*, 15–50.

(8) Kresse, G.; Furthmüller, J. Efficient Iterative Schemes for Ab Initio Total-Energy Calculations Using a Plane-Wave Basis Set. *Phys. Rev. B* **1996**, *54* (16), 11169–11186.

(9) Klimeš, J.; Bowler, D. R.; Michaelides, A. Chemical Accuracy for the van Der Waals Density Functional. *J. Phys. Condens. Matter* **2010**, *22* (2), 022201. https://doi.org/10.1088/0953-8984/22/2/022201.

(10) Klime, J.; Bowler, D. R.; Michaelides, A. Van Der Waals Density Functionals Applied to Solids. *Phys. Rev. B* **2011**, *83* (19), 195131. https://doi.org/10.1103/PhysRevB.83.195131.

(11) Wellendorff, J.; Lundgaard, K. T.; Møgelhøj, A.; Petzold, V.; Landis, D. D.; Nørskov, J. K.; Bligaard, T.; Jacobsen, K. W. Density Functionals for Surface Science: Exchange-Correlation Model Development with Bayesian Error Estimation. *Phys. Rev. B* **2012**, *85* (23), 235149. https://doi.org/10.1103/PhysRevB.85.235149.

(12) Sharp, J.; Ciotti, A.; Andrews, H.; Udayasurian, S. R.; García-Melchor, M.; Li, T. Sustainable Electrosynthesis of Cyclohexanone Oxime through Nitrate Reduction on a Zn-Cu Alloy Catalyst. *ACS Catal.* **2024**, *14* (5), 3287–3297. https://doi.org/10.1021/acscatal.3c05388.

(13) Ciotti, A.; García-Melchor, M. The Importance of Surface Coverages in the Rational Design of Electrocatalysts. *Curr. Opin. Electrochem.* **2023**, *42*, 101402. https://doi.org/10.1016/j.coelec.2023.101402.

(14) Blöchl, P. E. Projector Augmented-Wave Method. *Phys. Rev. B* **1994**, *50* (24), 17953–17979.

(15) Methfessel, M.; Paxton, A. T. High-Precision Sampling for Brillouin-Zone Integration in Metals. *Phys. Rev. B* **1989**, *40* (6), 3616–3621.

(16) Hjorth Larsen, A.; Jørgen Mortensen, J.; Blomqvist, J.; Castelli, I. E.; Christensen, R.; Dułak, M.; Friis, J.; Groves, M. N.; Hammer, B.; Hargus, C.; Hermes, E. D.; Jennings, P. C.; Bjerre Jensen, P.; Kermode, J.; Kitchin, J. R.; Leonhard Kolsbjerg, E.; Kubal, J.; Kaasbjerg, K.; Lysgaard, S.; Bergmann Maronsson, J.; Maxson, T.; Olsen, T.; Pastewka, L.; Peterson, A.; Rostgaard, C.; Schiøtz, J.; Schütt, O.; Strange, M.; Thygesen, K. S.; Vegge, T.; Vilhelmsen, L.; Walter, M.; Zeng, Z.; Jacobsen, K. W. The Atomic Simulation Environment - A Python Library for Working with Atoms. *J. Phys. Condens. Matter* **2017**, *29* (27), 273002. https://doi.org/10.1088/1361-648X/aa680e.

(17) Jain, A.; Ong, S. P.; Hautier, G.; Chen, W.; Richards, W. D.; Dacek, S.; Cholia, S.; Gunter, D.; Skinner, D.; Ceder, G.; Persson, K. A. Commentary: The Materials Project: A Materials Genome Approach to Accelerating Materials Innovation. *APL Mater.* **2013**, *1* (1), 011002. https://doi.org/10.1063/1.4812323.

(18) Birch, F. Finite Elastic Strain of Cubic Crystals. *Phys. Rev.* **1947**, *71* (11), 809–824.

(19) Wang, V.; Xu, N.; Liu, J. C.; Tang, G.; Geng, W. T. VASPKIT: A User-Friendly Interface Facilitating High-Throughput Computing and Analysis Using VASP Code. *Comput. Phys. Commun.* **2021**, *267*, 108033. https://doi.org/10.1016/j.cpc.2021.108033.

(20) Nørskov, J. K.; Rossmeisl, J.; Logadottir, A.; Lindqvist, L.; Kitchin, J. R.; Bligaard, T.; Jónsson, H. Origin of the Overpotential for Oxygen Reduction at a Fuel-Cell Cathode. *J. Phys. Chem. B* **2004**, *108* (46), 17886–17892. https://doi.org/10.1021/jp047349j.

(21) Nørskov, J. K.; Bligaard, T.; Logadottir, A.; Kitchin, J. R.; Chen, J. G.; Pandelov, S.; Stimming, U. Trends in the Exchange Current for Hydrogen Evolution. *J. Electrochem. Soc.* **2005**, *152* (3), J23–J26. https://doi.org/10.1149/1.1856988.

(22) Groß, A. Reversible vs Standard Hydrogen Electrode Scale in Interfacial Electrochemistry from a Theoretician's Atomistic Point of View. *J. Phys. Chem. C* **2022**, *126* (28), 11439–11446. https://doi.org/10.1021/acs.jpcc.2c02734.

(23) Gossenberger, F.; Roman, T.; Groß, A. Hydrogen and Halide Co-Adsorption on Pt(111) in an Electrochemical Environment: A Computational Perspective. *Electrochim. Acta* **2016**, *216*, 152–159. https://doi.org/10.1016/j.electacta.2016.08.117.
